# Supplementary material for: Globally unequal effect of extreme heat on economic growth
Source: Sci Adv. 2022 Oct 28;8(43):eadd3726. doi: 10.1126/sciadv.add3726 (PMC9616493; doi:10.1126/sciadv.add3726)
Supplement: 20221028-1 [file sciadv.add3726.v1.pdf]

ECONOMICS

Globally unequal effect of extreme heat on economic growth

Copyright © 2022 The Authors, some rights reserved; exclusive licensee American Association for the Advancement of Science. No claim to original U.S. Government Works. Distributed under a Creative Commons Attribution License 4.0 (CC BY).

Christopher W. Callahan[1,2]* and Justin S. Mankin[2,3,4]

Increased extreme heat is among the clearest impacts of global warming, but the economic effects of heat waves are poorly understood. Using subnational economic data, extreme heat metrics measuring the temperature of the hottest several days in each year, and an ensemble of climate models, we quantify the effect of extreme heat intensity on economic growth globally. We find that human-caused increases in heat waves have depressed economic output most in the poor tropical regions least culpable for warming. Cumulative 1992–2013 losses from anthropogenic extreme heat likely fall between $5 trillion and $29.3 trillion globally. Losses amount to 6.7% of Gross Domestic Product per capita per year for regions in the bottom income decile, but only 1.5% for regions in the top income decile. Our results have the potential to inform adaptation investments and demonstrate how global inequality is both a cause and consequence of the unequal burden of climate change.

INTRODUCTION

Increases in extreme heat from anthropogenic global warming (*1*, *2*) pose alarming risks to human well-being (*3*, *4*). These risks are particularly acute in the poorest and warmest regions on Earth, located in the tropics, where changes in the tails of the temperature distribution have emerged first (*5*–*7*). Because of their warmth, tropical regions are at risk to cross physiological temperature thresholds for human morbidity and mortality (*3*, *8*). Moreover, lower incomes make tropical economies less able to adapt to increases in extreme heat (*9*). Even modest increases in mean temperatures can cause large increases in extremes (*10*–*13*), so increased heat extremes due to warming will stress adaptive capacities in the low-income regions that have contributed least to climate change (*14*, *15*).

There is a long history of studying, documenting, and predicting extreme heat events given the risks they pose to people (*1*, *2*, *13*) and their nonlinear response to forcing (*10*, *16*). However, despite the centrality of extreme heat to the human experience of the climate system—as well as to present and future climate impacts—there has been little quantification of its macroeconomic costs. Without knowing the scope of economic losses from heat waves, it is difficult to conceive risk management and preparedness strategies that are commensurate with their costs. Part of this gap emerges from data realities: Extreme heat occurs at fine spatiotemporal scales, imposing strict requirements on the resolution and extent of meteorological and socioeconomic data. To overcome these data limitations, climate-economy studies tend to focus on small regions of high-quality data (*17*, *18*) or relate global average temperatures to economic losses using functions that assume local risks are captured by mean changes (*19*). Crucially, however, these loss functions have not been well constrained by empirical estimates of the effects of extreme events (*20*).

Empirical research has shown that extreme temperatures reduce labor productivity (*21*), damage crops (*22*, *23*), and increase mortality (*9*), among other effects. Because this research is often sector or region specific (*24*–*26*), a theoretical and empirical gap still remains between the nonlinearities identified at the local and sectoral level and the global economic assessments required to evaluate differing climate policies. Several empirical climate-economy studies have quantified the global economic effects of changes in average temperature (*27*–*32*) and temperature variability (*33*) in an attempt to close this gap. However, the physical processes driving average and extreme temperatures are fundamentally distinct (*34*, *35*). Extreme heat events are driven by atmospheric blocking events (*36*, *37*) and land-atmosphere feedbacks, such as soil drying, that can amplify the anticyclonic circulation patterns required for multiday heat accumulation (*38*, *39*). These processes take place on characteristic daily-to-weekly time scales and have length scales associated with the synoptic or finer. While related, these processes are not the same as those that determine climatological quantities such as annual mean temperature. Furthermore, because anthropogenic warming is projected to warm the hottest days of the year more than annual mean temperatures due to land-atmosphere feedbacks (*10*, *40*, *41*), assessing the unique economic effects of the hottest few days of the year is necessary to more fully quantify the costs of global warming.

The goal of our work is to directly quantify the global macroeconomic effects of extreme heat, to identify the risks associated with intensifying heat waves in a warming world, and to close a key gap in climate-economic research. As noted above, the requirement of high-resolution economic and disaster data challenges this effort. Data limitations are most acute in the world's poorest and warmest regions, which has skewed climate impacts research toward high-income areas (*17*, *18*). Data limitations are particularly important in the context of extreme heat because heat waves are often subnational in scale (*42*), complicating the country-level approach used in many empirical climate-economy studies (*30*, *31*).

Here, we empirically estimate the effect of extreme heat intensity on observed economic growth using data from a global sample of subnational regions. We measure extreme heat using the temperature of the hottest 5-day period in each year ("Tx5d"), although we assess several other metrics and find little difference in their effects. Tx5d and related metrics that measure the intensity of the hottest period of each year have been used in recent work to characterize changes in extreme heat (*1*, *11*, *43*–*45*) and have the benefit of capturing damaging multiday periods of extreme heat (*23*) while avoiding the arbitrary thresholds used in other heat wave metrics (*46*, *47*).

[1]Graduate Program in Ecology, Evolution, Environment and Society, Dartmouth College, Hanover, NH, USA. [2]Department of Geography, Dartmouth College, Hanover, NH, USA. [3]Department of Earth Sciences, Dartmouth College, Hanover, NH, USA. [4]Ocean and Climate Physics, Lamont-Doherty Earth Observatory of Columbia University, Palisades, NY, USA.
*Corresponding author. Email: christopher.w.callahan.gr@dartmouth.edu

Tx5d does not explicitly measure the frequency of extreme heat nor variations in its duration, so our identification strategy centers on measuring the effect of the intensity of the most extreme heat wave in each year. Other metrics can be used to assess these other characteristics (*13*, *46*, *47*) but can involve specifying relatively arbitrary temperature thresholds or bins, often at the cost of transparency and simplicity.

Our empirical model includes the effects of both Tx5d and annual average temperature, their interaction to allow the effect of extremes to vary with average temperature, and temperature variability (see Materials and Methods). This strategy allows us to identify the plausibly causal effect of extreme heat intensity while allowing different regions to respond differently on the basis of their annual average temperatures. It also allows us to consider how these three moments of the temperature distribution independently and jointly shape economic costs. We then combine these empirical results with historical climate model simulations to assess the economic effects of anthropogenic changes in extreme heat intensity to date (fig. S1).

RESULTS

Economic effects of extreme heat

Extreme heat significantly decreases economic growth in warm regions, weakly affects growth in temperate regions, and increases growth in cold regions (Fig. 1). For example, in Brazil, where the average temperature is a warm 23.8°C, an SD increase in Tx5d intensity depresses growth by 0.63 percentage points (p.p.). In contrast, in Norway, where the temperature averages 4°C, an additional SD in Tx5d intensity enhances growth by 0.62 p.p. (Fig. 1A). These results align with previous work, as the annual mean temperature at which extreme heat becomes harmful is ~14°C, similar to the temperature optima shown in studies of annual mean temperature (*27*, *28*).

The effects of extreme heat must be inferred for regions for which economic data is unavailable (hatching in Fig. 1B), which are primarily warm and low-income regions (Fig. 1A, histogram). Understanding the economic response to climate change is most useful when it is globally generalizable (*20*), motivating recent studies to extrapolate global climate-economy response functions from limited data (*9*, *31*). Here, we follow this practice by inferring the effect of extreme heat for all regions based on their average temperatures, even where economic data are unavailable (see the Supplementary Materials). We note that the sample on which we base our estimate covers ~66% of the world's population, spans regions with average temperatures that exceed 30°C, and encompasses tropical regions in countries such as Brazil, Indonesia, and India, suggesting that our model likely provides a reliable basis from which to infer the effect of extreme heat in both cool and warm regions.

Extrapolation of our results to regions without economic data emphasizes the latitudinal structure of the effect of extreme heat (Fig. 1B). Tropical regions lose income when extreme heat increases, mid-latitude regions in the United States and southern Europe lie in a weakly affected transition zone, and high-latitude regions gain economically as their baseline temperatures are too cold for optimal growth. Critically, warm tropical regions both tend to have lower incomes (Fig. 1A, top bar plot) and suffer the most from increased extreme temperatures. These low-income tropical regions are also the regions for which the least data are available (histograms in Fig. 1A), so this extrapolation procedure is a key limitation of our analysis. Gathering additional economic data in the regions most prone to climate impacts given their geography and income is an important focus for better attribution of climate impacts and therefore management of future climate risks.

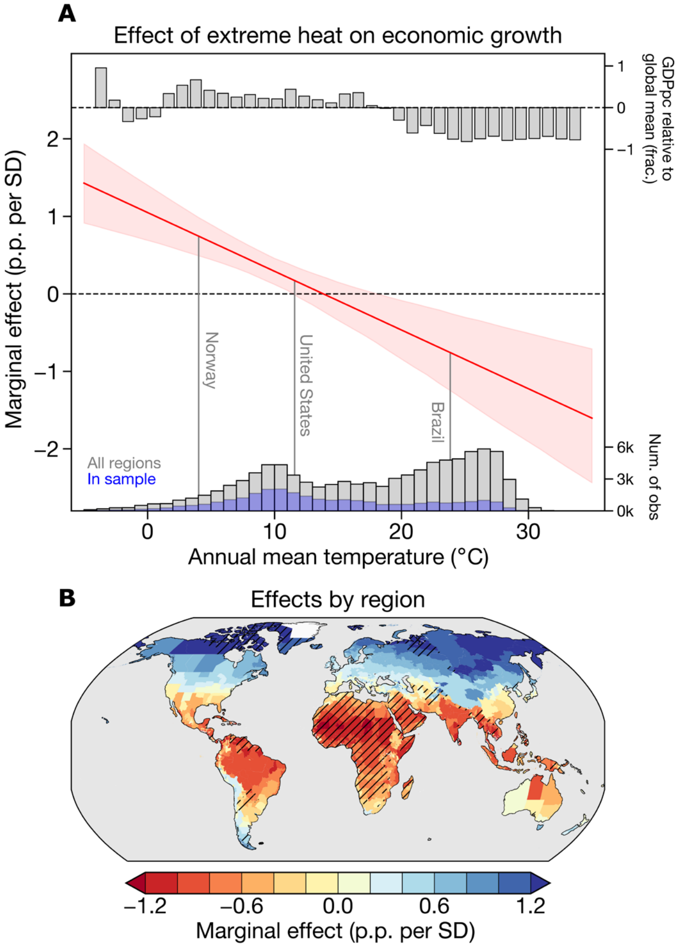

Fig. 1. Contemporaneous effect of extreme heat intensity on economic growth. (**A**) Marginal effects of extreme heat on economic growth in percentage points per SD of Tx5d (p.p. per SD) across a range of temperatures. Solid red line shows average estimates across 1000 bootstrap iterations, and shading shows 95% confidence intervals (see Materials and Methods). Vertical lines show the average temperatures for regions within selected countries. Top bar plot shows the average regional Gross Domestic Product per capita (GDPpc) in each 1°C temperature bin relative to the global mean regional GDPpc. Bottom histogram shows the distribution of temperatures in the estimation sample (blue) and all observations (obs) (gray). (**B**) Marginal effects for each region based on their average temperatures; hatching denotes regions where economic data are unavailable. Marginal effects are scaled by the average within-region SD of Tx5d (see Materials and Methods).

The effect of extreme heat on growth is robust to more restrictive standard error clustering and the addition of region-specific growth trends to control for time-varying unobservable factors (see Materials and Methods and table S1). Including region-specific trends is a useful way of controlling for time-varying cofounders but may lead to overfitting (*48*), especially given that some regions have fewer than 10 years of data (*33*), so we do not include trends in our main model. We quantify regression uncertainty via bootstrapping (see Materials and Methods), which also ensures that individual regions are not disproportionately driving the result. A placebo test, where we randomize extreme heat exposure and reestimate the model,

demonstrates that spurious trends across time or space are not driving our results (fig. S2). Including a squared precipitation term, as has been used in other recent studies (*27*, *30*), does not alter our results (table S1).

Our results are very similar when using other metrics of extreme heat, such as the intensity of the hottest 3-, 7-, or 15-day periods, although the peak 5-day period yields the strongest response (fig. S3). Using the temperature of the hottest day or the hottest month yields weaker and more uncertain responses (fig. S3), as these metrics either do not capture multiple days of exposure (e.g., hottest day) or average temperatures over an excessively long period (e.g., hottest month). The 5-day time period associated with the peak effect of extreme heat is physically consistent with synoptic time scales; heat waves are generally driven by large-scale high-pressure systems (*37*), which evolve on the daily-to-weekly time scales associated with continental-scale atmospheric circulation.

Our use of annual mean temperature as the interaction variable with Tx5d implies that heterogeneity in the effect of extreme heat is driven only by variation in underlying average temperatures. We interpret this heterogeneity as being driven primarily by climatological temperature as opposed to interannual variation in annual average temperature, because within-region variation in annual average temperature is an order of magnitude smaller than across-region variation (the overall SD of annual average temperature across our sample is 7.7°C, but the average within-region SD is only 0.52°C). This interpretation is supported by a model in which we interact Tx5d with long-term regional average temperature rather than annual mean temperature and find very similar results (table S2).

Temperature covaries with other variables that may drive heterogeneity in the effect of extreme heat: Warmer regions also generally have lower income (Fig. 1A), and warmer years may also be drier, so income and drought may be additional moderators of the effect of extremes. To test these hypotheses, we estimate additional models with average income and the Palmer Drought Severity Index as the interaction terms (table S2). Neither of these variables have significant interactions with Tx5d, and when the annual mean temperature interaction is added alongside these variables, only the temperature interaction is significant (table S2). These results support the interpretation that temperature is the primary moderator of the effect of extreme heat, consistent with earlier work that has found differences in the effect of warming to be due to differences in temperature exposure, not differences in income (*27*, *30*).

Our findings reinforce the fact that people are poorly adapted to extreme heat in the present day, even in regions inured to being warm. Many adaptations have been undertaken to cope with extremely hot conditions independent of climate change. In high-income regions, this often takes the form of air conditioning for indoor spaces (*49*) alongside a broader shift to service-dominated economies (*50*). In low-income regions, adaptations are primarily behavioral (*51*), including resting in the shade, drinking more water, and shifting to nonoutdoor labor when possible (*52*). However, there are physiological thresholds for extreme heat exposure in people (*3*) and agriculture (*22*) that challenge the efficacy of behavioral adaptations. Our results demonstrate that current adaptations have not been successful in eliminating the negative effects of extreme heat and emphasize the need for further such adaptation investments alongside climate mitigation.

Our empirical model controls for mean temperatures to assess how growth is differentially affected by average temperatures and extremes. Previous studies have argued that mean temperatures should capture the effect of extremes (*27*). However, the geophysical processes driving average temperatures are different from those driving extremes (*34*, *35*). Furthermore, because temperature distributions are often non-Gaussian and can have long tails (*53*), the relationship between average and extreme temperatures is complex and warrants further study (*54*). Annual average temperatures explain ~40% of the variation in raw Tx5d values in our sample (fig. S4), but this occurs in large part because regions that are warmer, on average, have both greater annual temperatures and greater Tx5d values. When expressed as deviations from regional means, which is consistent with our estimation with the fixed effects model (i.e., purging time-invariant regional characteristics; see Materials and Methods), annual average temperature anomalies explain less than 13% of the variation in Tx5d anomalies (fig. S4). Hence, there is substantial variation in extreme temperatures that might harm growth not captured by models that only consider annual average temperature. The value of incorporating Tx5d relative to a model that only includes annual average temperature is supported by a likelihood ratio test, which indicates that the model that includes Tx5d has greater explanatory power than the model that does not ($P < 0.0001$).

Our results show significant independent effects of both average and extreme temperatures. Increases in average temperatures, for example, have weakly positive effects in cold regions and increasingly harmful effects in warmer regions (fig. S5). This pattern is consistent with recent work on the economic impacts of increasing average temperatures (*27*, *30*, *31*). However, we find that the squared mean temperature term is no longer statistically significant, while the interaction between mean temperature and Tx5d is significant (table S1). This result implies that increases in mean temperatures are harmful in warmer locations, and this additional harm arises because of interactions between warmer mean temperatures and increased extreme heat intensity in the warmest part of the year.

Following recent work, our model also includes the effect of daily-scale temperature variability (see Materials and Methods) (*33*). We find a strong negative effect of temperature variability on growth (fig. S5), consistent with Kotz *et al.* (*33*). One key question is whether increases in variability are intrinsically damaging or only damaging insofar as they induce greater temperature extremes. Our finding of a negative effect of variability (−2.01 p.p. per SD) in a model that includes Tx5d implies that variability is indeed intrinsically damaging. However, when we remove the Tx5d terms from the model, the variability effect increases in magnitude to −2.21 p.p. per SD. That difference demonstrates that when extreme heat is not explicitly considered, it is possible to find an effect of temperature variability that is 10% too large because years with greater variability also imply more extreme temperatures in the hottest parts of the year.

The spatial patterns of economic damages from both average temperature and variability are similar: Effects are negative everywhere but largest in warm and low-variability tropical regions, which amplifies the unequal effects of global warming. In contrast, extreme heat has a distinct spatial pattern. Extreme heat causes large damages in low-latitude regions and transitions to modest damages and lastly benefits as latitude increases (fig. S5). The fact that extreme heat exhibits an effect that reverses with latitude, while average temperatures and variability do not, means that analyzing temperature extremes is necessary to fully account for the global inequities in the burden of temperature impacts.

Anthropogenic increases in extreme heat

These empirical findings imply that human-caused warming has affected economic production through changes in extreme heat. Estimating how much anthropogenic extreme heat has affected global economic production requires three things: (i) the effect of extreme heat on economic growth; (ii) anthropogenic changes in extreme heat; and (iii) continuous Gross Domestic Product per capita (GDPpc) data (see Materials and Methods). Our empirical model provides the first of these. To estimate the second, we use historical and natural climate model experiments from the sixth phase of the Coupled Model Intercomparison Project (CMIP6) (table S3) (*55*, *56*) to calculate "counterfactual" Tx5d. To ensure the availability of continuous GDPpc data to meet the third criterion, we use a statistical model to infer regional GDPpc time series over 1992–2013 where they are not currently available (see Materials and Methods).

Anthropogenic warming has increased the frequency and intensity of extreme heat events globally, but the spatial pattern is heterogeneous, increasing most strongly in the tropics (Fig. 2). Globally, regional Tx5d values average 0.77°C more than they would have without warming over 1992–2013, with increases of more than 1°C in much of the tropics but less than 0.5 in the United States and Europe (Fig. 2A). The probability of extreme Tx5d values (the 90th percentile in each region calculated from the counterfactual time series) has also substantially increased, with probabilities rising by 13 p.p. across regions, on average, and even more intensely across South America, Africa, and the Middle East (Fig. 2B). In contrast, the probabilities of 90th percentile Tx5d values have risen less than 5 p.p. or even decreased in much of the midlatitudes. The spatial pattern of anthropogenic extreme heat thus coincides with both the stronger economic effect of extreme heat in the tropics and the lower incomes there, making tropical regions particularly vulnerable to losses from human-caused heat extremes.

Economic recovery from extreme heat

Quantifying the total economic output change attributable to anthropogenic extreme heat requires knowledge of whether regions recover from extreme heat and how long this recovery takes. If extreme heat affects income levels, but not growth, then economies will "catch up" following a heat wave, recovering to their previous income trajectory. Destroyed crops may be resown in the time after a heat wave, for example, and investment may flow into damaged areas (*57*). If, instead, extreme heat affects the underlying ability of the economy to grow, then damages could compound over the long run (*58*), permanently altering a region's income trajectory. Permanent growth effects can generate large uncertainties when projected into the future (*48*), which means empirically testing the persistence of the effect of extreme heat is a critical task in climate damage calculations. Hence, we estimate a distributed lag version of our empirical model, which allows us to track the persistence of the impacts of a heat wave in the years after it occurs (see the Supplementary Materials).

The effect of extreme heat intensifies in the first year after the event and can persist for an additional year, before becoming indistinguishable from zero in the second or third year after the event (Fig. 3A and fig. S6). In cold regions (e.g., annual average temperatures of 5°C), the positive effect of extreme heat converges to zero in the second year after the event and even becomes negative after that, although these negative effects are not statistically significant (Fig. 3A, blue lines). In warm regions (e.g., 25°C), the cumulative effect of extreme heat nearly doubles in the year following the event but converges to zero in the two years after that (Fig. 3A, red lines), suggesting a delayed rebound effect. Specifying an autoregressive distributed lag model with up to four autoregressive terms, following recent work (*32*, *57*), yields very similar results (fig. S7).

These dynamics are characteristic of a prolonged, multiyear level effect, as regions that were harmed can rebuild damaged capital or resume previous productivity in the time after the heat wave (Fig. 3B). However, the delay of several years means that regions lose or gain multiple years of income in response to a heat wave (Fig. 3C). Even if regions return to their original income trajectory after a heat wave, there remains a gap between their actual income and the income they would have experienced without the heat wave (Fig. 3C). Therefore, while individual heat extremes do not permanently affect economic growth, repeated extreme heat events might generate tangible long-term income changes.

Global economic effects of anthropogenic extreme heat

To assess the economic impacts of anthropogenic changes in extreme heat, we apply the distributed lag model coefficients (Fig. 3A) to observed and counterfactual Tx5d values. We also use observed and counterfactual average temperatures in this calculation, meaning that we incorporate both changes in extreme heat and in the underlying average temperatures that shape the marginal effect of

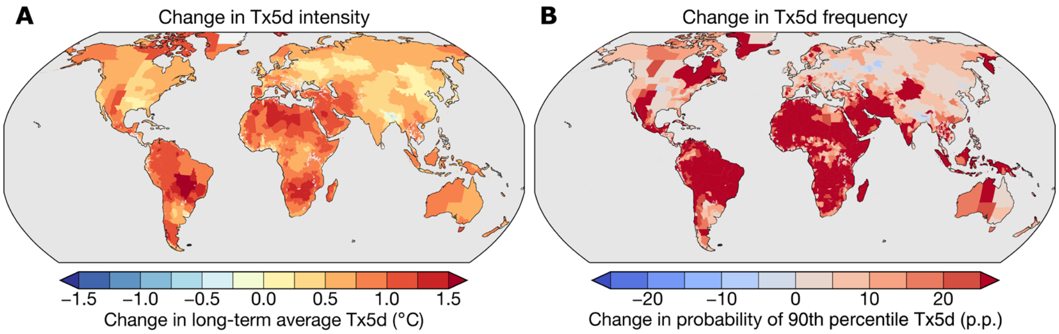

Fig. 2. Anthropogenic changes in extreme heat. (**A**) Ensemble mean change in each region's average Tx5d value between the observed and counterfactual climates estimated using CMIP6 climate models. (**B**) Ensemble mean change in the probability of each region's counterfactual 90th percentile Tx5d value between the observed and counterfactual climates. Increases in both quantities imply that the values are higher in the observed climates than in the counterfactual climate. Ensemble mean values are calculated by first averaging over individual realizations within models and then averaging across models. Values are calculated over 1992–2013 to overlap with the period over which the damages calculations are performed.

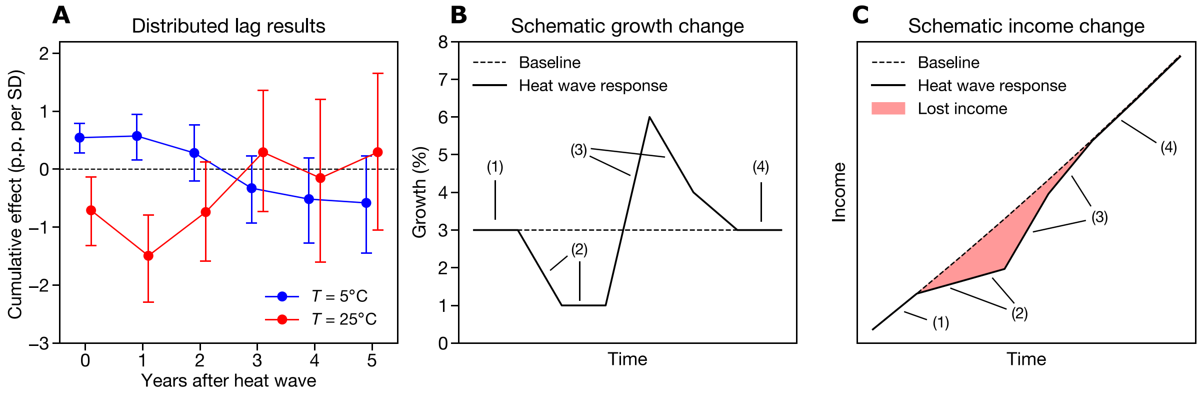

Fig. 3. Evolution of the growth response to extreme heat intensity. (**A**) Distributed lag coefficients for two example regions, one with an annual average temperature of 25°C (red) and one with an annual average temperature of 5°C (blue). Dots show averages, and bars show 95% confidence intervals from bootstrap resampling (see Materials and Methods). (**B**) Simulated response of annual GDPpc growth in a constant 3% growth baseline scenario (dashed line) and a heat wave scenario in a warm region (solid line). (**C**) Simulated response of annual GDPpc expressed as income change in a constant 3% growth baseline scenario (dashed line) and a heat wave scenario in a warm region (solid line). Shaded red area denotes the difference between the baseline and heat wave scenarios, representing the income lost due to the heat wave. Annotations in (B) and (C) represent the initial constant-growth period (1), the period in which growth declines because of the heat wave (2), the period in which growth rebounds and increases relative to the initial period (3), and the period in which growth stabilizes back to its initial rate (4).

extreme heat (see the Supplementary Materials). The result is a time series of the change in economic growth in each subnational region due to anthropogenic changes in Tx5d over 1992–2013. These time series can be calculated for all regions, even those without GDPpc data, since they only depend on the region's observed and counterfactual Tx5d and average temperature values.

These growth changes allow us to calculate time series of counterfactual regional GDPpc (*29*). Problematically, there are many subnational regions, especially in Africa and Southeast Asia, where regional GDPpc data are missing (hatched regions in Fig. 1B). To address this data gap, we use a simple statistical model to downscale country-level GDPpc to generate continuous time series of regional GDPpc (see Materials and Methods). We predict regional GDPpc using country-level GDPpc and regional nighttime luminosity, two predictors that have been previously shown to effectively predict local economic output (*59*–*61*). Our model skillfully reproduces regional GDPpc in our sample (R^2 = 0.895; fig. S8). *K*-fold cross-validation tests demonstrate that country-level GDPpc and regional nighttime luminosity have low out-of-sample prediction error and outperform models that include additional predictors such as regional area, population, or crop yields (fig. S9).

We then apply the growth change values calculated from the climate model simulations to generate counterfactual regional GDPpc. The difference between observed and counterfactual GDPpc represents the effect of anthropogenic extreme heat (see Materials and Methods). To quantify uncertainty in this procedure, we perform a Monte Carlo analysis, resampling uncertainty from the econometric model, the climate models, variability from the statistical model used to predict regional GDPpc, and residual uncertainty in regional GDPpc (see Materials and Methods).

Anthropogenic changes to extreme heat thus far have primarily harmed tropical regions (Fig. 4A). GDPpc is >5% per year lower than it would have been without anthropogenic effects on extreme heat in tropical countries such as Brazil, Venezuela, and Mali. In high-latitude nations such as Canada and Finland, anthropogenic extreme heat changes have depressed GDPpc by ~1% per year. While the damages we find here are smaller than previous estimates using average temperature alone (*29*), they are economically significant: cumulative losses over 1992–2013 total $39 billion (2010-equivalent dollars) in the average region in Brazil, more than half of the 2010 GDP in the average Brazilian region, and $6.5 billion in the average Indonesian region, >44% of the 2010 GDP in the average Indonesian region (Fig. 4B). Many high-income countries such as the United States have lost relatively little in relative terms but tens of billions in absolute terms due to their large economies. These effects are smaller than those found by Diffenbaugh and Burke (*29*) from annual mean temperature but are comparable to those found by Miller *et al.* (*23*) projecting future losses from heat waves (although Miller *et al.* focus primarily on agricultural output).

These region-level losses aggregate into a clear global picture: Cumulative global losses due to extreme heat average more than $16 trillion in 2013 (Fig. 4C). On the basis of our uncertainty analysis and Intergovernmental Panel on Climate Change uncertainty terminology (*62*), it is virtually certain that the cumulative global effects of extreme heat fall between losses of $65 trillion and gains of $16 trillion, very likely that they fall between losses of $43 trillion and gains of $1.1 trillion, and likely that they fall between losses of $29.3 trillion and $5 trillion (Fig. 4C).

Despite the clear signal of extreme heat in global income, the global picture belies substantial inequalities in the magnitude of damages at the regional level (Fig. 4, D and E). Regions with greater income per capita are more likely to experience benefits or limited damages from human-caused changes in heat extremes, while regions with lower income per capita experience greater relative damages (Fig. 4D). For example, regions in the lowest income decile have experienced losses of 6.7% of GDPpc per year, compared to 1.5% in the top income decile. Moreover, while our Monte Carlo analysis (see Materials and Methods) highlights substantial uncertainty in the total global effect of extreme heat (Fig. 4C), uncertainty in individual regions is often low (Fig. 4D). In more than half of regions, primarily located in the tropics, more than 90% of Monte Carlo simulations result in economic losses rather than benefits. In the lower five income deciles, even the 99% range does not encompass zero (Fig. 4D). Thus, even in regions where uncertainty about observed

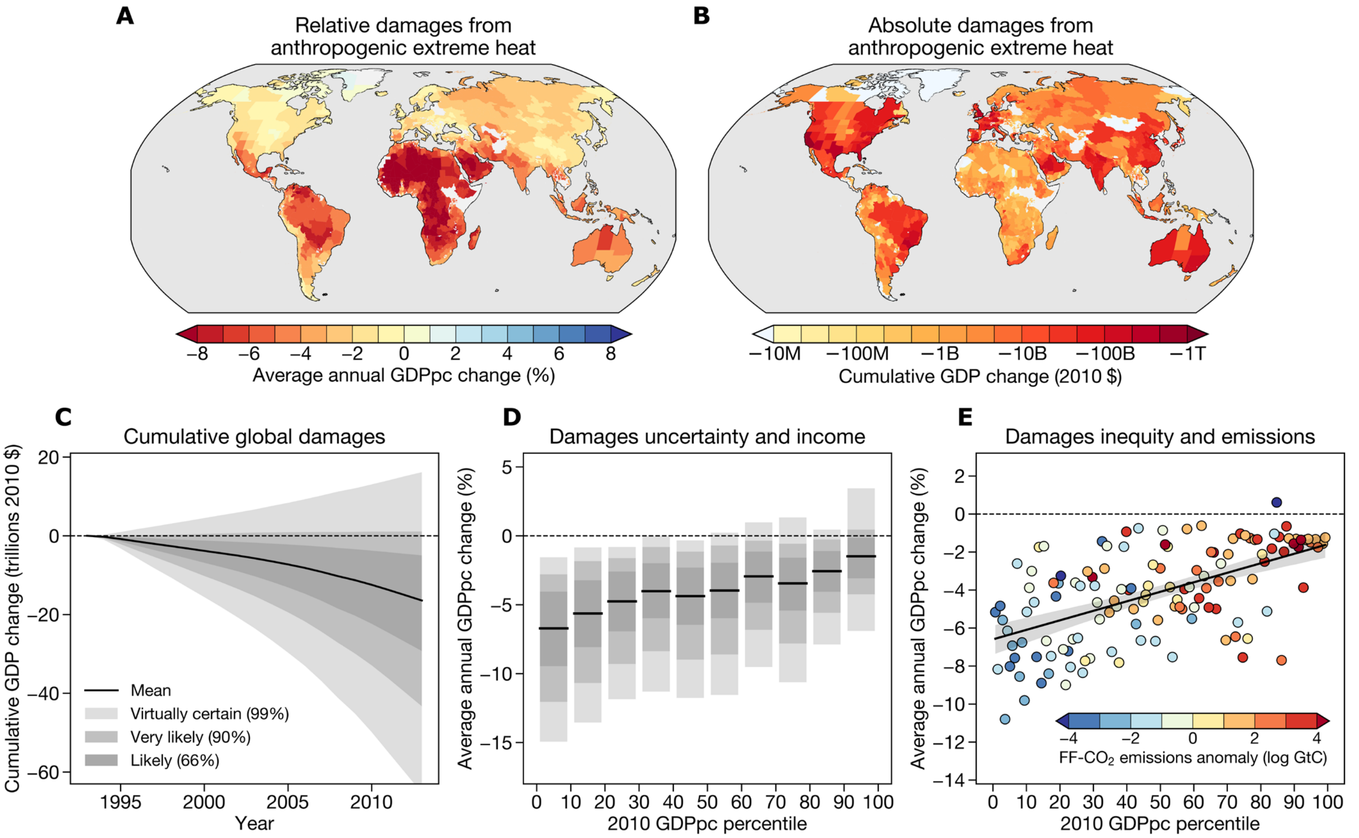

Fig. 4. Unequal economic effects of anthropogenic changes to extreme heat intensity. (**A**) Average annual change in regional GDPpc due to anthropogenic changes in Tx5d intensity over 1992–2013. (**B**) Cumulative 1992–2013 change in regional GDP in 2010 U.S. dollars due to anthropogenic changes in Tx5d intensity. (**C**) Cumulative global GDP change due to anthropogenic changes in extreme heat. Black line denotes the mean across 10,000 Monte Carlo simulations, and gray shading denotes the Intergovernmental Panel on Climate Change likely (66%), very likely (90%), and virtually certain (99%) ranges. (**D**) Average annual change in regional GDPpc due to anthropogenic extreme heat, binned by regional income deciles. Uncertainty visualization is the same as (C). (**E**) Relationship between each country's 2010 GDPpc percentile and the regional-average effect of anthropogenic changes to extreme heat in that country. Colors denote each country's fossil fuel CO_2 (FF-CO_2) emissions anomaly (difference between its log cumulative FF-CO_2 emissions and global mean log cumulative FF-CO_2 emissions). Color bar units are the log of gigatons of carbon (GtC). Black line is the least-squares regression line with the 95% confidence intervals shaded.

economic output is high, we can be virtually certain that this output has been reduced by anthropogenic increases in extreme heat.

Poorer regions have higher baseline temperatures and lower temperature variability, so those regions both experience the signal of extreme heat from warming first (*5*–*7*) and suffer most when extreme heat increases. Previous work has tied climate change to increased global economic inequality (*29*), and our results strengthen these findings. However, the inequality of climate change extends to its causes, not just its effects. Rich countries that experience limited damages are also large emitters of fossil fuel carbon dioxide (CO_2), making them primarily responsible for increases in global temperatures and associated heat extremes (Fig. 4E). Given the strong relationship between cumulative CO_2 emissions and changes in local temperature extremes (*63*), high-emitting nations can be considered directly responsible for a large fraction of warming-induced heat extremes and, by extension, the income losses suffered by individual regions (*64*).

The spatial pattern of the damages we find is clearly different from the original pattern of the marginal effect of extremes (cf. Figs. 4A and 1B): High-latitude regions benefit from extreme heat in our empirical setting but have suffered losses by anthropogenic forced changes in extreme heat to date. The discrepancy occurs because our damages calculation incorporates changes in mean temperatures along with extremes, so climate change has modified the marginal effect of extremes alongside their magnitude and frequency. Warmer annual mean temperatures make extremes more harmful (Fig. 1), so anthropogenic increases in average temperatures make extreme heat become damaging even where it originally appeared beneficial. Alternatively, if we hold average temperatures constant at their observed values, then damages are substantially reduced in magnitude (2 to 4% GDPpc losses in tropical regions), and the spatial pattern more closely resembles the pattern of marginal effects in Fig. 1B (fig. S10).

Here, we focus primarily on the historical effects of extreme heat since its economic effect is unknown. Although climate change has myriad other impacts, specifically quantifying the effects of increased intensity in the hottest 5 days of the year allows decision-makers to weigh the benefits of adaptations focused on those few days. However, we also calculate damages from changes in average temperatures and variability to compare them to those from changes in extremes (see the Supplementary Materials). Historical changes in average temperatures have caused uniform losses globally, with greater magnitudes than the losses from extreme heat (fig. S11). This is because

the effects of average temperatures appear to persist (fig. S12), rather than being recovered similar to the effects of extreme heat, consistent with previous findings (*27*, *30*). Human-caused changes in temperature variability are heterogeneous (*65*), and the effects of variability are recovered after 4 years (fig. S12). Hence, damages from anthropogenic changes in variability are spatially complex and smaller than the damages from extreme or average temperatures (fig. S11). When combined, damages associated with all three variables appear primarily driven by changes in average temperatures (fig. S11). However, changes in extreme heat intensity account for almost 10% of the overall damages despite being based on <2% of days in the year, indicating that the hottest few days of the year have an outsize influence on the economic effects of warming.

DISCUSSION

Our work yields three key results: (i) Increased extreme heat intensity significantly decreases economic growth in relatively warm tropical regions and weakly affects it in relatively cool midlatitude regions; (ii) anthropogenic climate change has increased the frequency and intensity of these economically consequential heat extremes; and, therefore, (iii) the effects of climate change on extreme heat have amplified underlying inequality, disproportionately harming low-income, low-emitting regions, with major emitters shouldering primary responsibility for billions of dollars of losses in the tropics. These results show that the local and sectoral nonlinear effects of extreme heat integrate into a coherent and global macroeconomic response, helping to close a key gap in climate-economic research. They also emphasize that, while related, the costs of temperature extremes are distinct from the costs of average temperatures and temperature variability. Hence, the true economic costs of temperature changes depend on which moment of the temperature distribution an analysis considers.

These results complement and extend existing work that shows the negative economic effects of heat exposure. Miller *et al.* (*23*) showed that consecutive hot days exceeding location-specific thresholds weakly reduce growth in country-level overall GDP and strongly reduce growth in country-level agricultural GDP and that these effects are stronger in warmer years. Our work is consistent with theirs, showing negative effects of multiday periods of extreme heat on economic growth. However, two distinctions between our work and theirs are worth noting. First, we identify a stronger signal in overall GDP growth because we account for the subnational spatial scale of extreme heat, rather than aggregating to the country scale. Second, while the index designed by Miller *et al.* (*23*) usefully captures cumulative short-term exposure to extreme heat, it relies on user-defined location-specific thresholds and produces discontinuities directly above and below those thresholds. Our work extends theirs by showing a strong effect of extreme heat with a widely used and transparent index of heat intensity.

A complementary approach to measuring extreme heat is to count days falling into a set of temperature bins, which also reveals negative effects of extreme heat (*21*, *25*, *30*). However, existing implementations of the binning approach specify absolute temperature bins, which do not account for variation in what counts as "extreme." A day that is mild by the standards of Nigeria may be extreme by the standards of Norway. Acclimation to the local temperature distribution has been shown to manifest in, for example, the temperature-mortality relationship (*9*) but is not considered by most implementations of the binning approach. Using the temperature of the hottest heat wave in each region provides a more flexible way to measure the effect of temperatures that are extreme relative to the local climate.

Last, the spatial pattern of damages we find is similar to other studies that have focused on annual mean temperature, such as Burke *et al.* (*27*) and Diffenbaugh and Burke (*29*). These studies have generally found net benefits at high latitudes from mean warming instead of weaker losses, as we find here with extremes (Fig. 4A). However, a consistent result across studies is that tropical regions will suffer most because of their high baseline temperatures. Thus, an important insight from our work that extends previous analyses is that some of the economic damages arising from changes in average temperatures are actually due to increases in the intensity of the hottest few days in each year.

Our results are subject to two key caveats. First, we focus on extreme heat intensity as opposed to the potentially distinct effects of extreme heat frequency or duration. Second, there are considerable data limitations in the world's hottest and lowest income regions.

First, Tx5d measures the temperature of the hottest 5-day heat wave in each year but does not explicitly incorporate information about additional, less hot periods of extreme heat or interannual variations in heat wave duration. Measuring the integrated effect of all periods of extreme heat within a given year would require a temperature threshold for what counts as extreme. While emerging work has begun to examine the effect of crossing location-specific thresholds in the context of extreme temperature (*23*) and rainfall (*50*), the choice of threshold is generally arbitrary and poorly constrained in the context of macroeconomic growth, so we focus on the peak intensity of extreme heat due to its simplicity and clarity. Multiple periods of extreme heat may have compounding and nonlinear effects (*23*), so incorporating additional periods would likely enhance the effect of extreme heat. Hence, our results should be viewed as conservative. In addition, Tx5d and similar metrics measuring the hottest several-day period in each year have been widely used in the physical science literature on extreme heat (*1*, *11*, *43*–*45*), making them an appropriate metric for use in integrating physical climate projections of anthropogenic extreme heat with econometric estimates of its macroeconomic effect.

Second, our analysis is limited by missing data in many of the world's poorest and hottest regions, such as Africa (Fig. 1A). Subnational economic data are limited in these regions, but using subnational data to measure the effect of extreme heat is an important contribution of our analysis, since heat waves occur at subnational spatial scales (*42*). As a result, our estimates of the effect of extreme heat in very hot regions go beyond the support of the data. However, our estimation sample covers 66% of the world's population, includes regions with annual temperatures up to 30.3°C, close to the maximum annual mean temperature of 31.7°C, and encompasses hot regions in countries such as Brazil, Indonesia, and India (Fig. 1A). The key assumption underlying our extrapolation procedure is that the underlying average temperature controls the marginal effect of extreme heat globally. This assumption is supported both by multiple functional forms testing alternative metrics of heterogeneity and by existing work demonstrating the role of underlying baseline temperatures in shaping the effect of rising temperatures (*9*, *27*, *66*). As additional data in regions such as Africa and Southeast Asia become available, they will allow us to further constrain the mechanisms that shape the economic effect of extreme heat globally.

Data limitations also make calculating accumulated economic damages difficult, which motivated our work to generate continuous, globally representative subnational income data (see Materials

and Methods). While these results are subject to further refinement as the science of measuring local well-being improves (*67*), our use of nighttime luminosity data is consistent with an emerging literature that uses these data to infer local economic production (*59*–*61*). In addition, our uncertainty propagation analysis (see Materials and Methods) allows us to responsibly account for the uncertainties introduced by each of these limitations. More broadly, allowing data limitations to prevent an analysis of economic damages from extreme heat in the world's hottest and poorest regions would be a disservice to the people in these regions, who are simultaneously most vulnerable to climate change, least culpable for it, and least well-represented by existing data (*17*, *18*).

Despite these caveats, our findings have important adaptation implications: Targeting resources at heat resilience and early-warning capabilities for only a few days per year may yield disproportionate economic benefits. These targeted benefits were not apparent in previous studies that have focused on annual mean temperatures. On one hand, many important infrastructural adaptations, such as air conditioning and expanded green spaces, could deliver major benefits but would be installed year-round instead of being tailored to the few hottest days in each year (*68*). On the other hand, contingent and temporary adaptations such as converting public spaces into cooling centers, deploying public evaporative cooling systems, and expanding emergency service availability could yield disproportionate benefits relative to their cost if deployed specifically during the hottest few days of the year. In addition, expanded implementation of heat early warning systems such as was implemented in France after the 2003 heat wave (*69*) could allow people to take measures such as reducing nonessential electricity usage to avoid blackouts and avoiding outdoor activities when possible.

That warming has already increased the frequency and intensity of heat extremes is well known, but our results demonstrate the economic costs of these events and their unequal global distribution. Our work therefore increases the urgency of both climate mitigation efforts and investments focused on increasing the adaptive capacity of the poorest parts of the world for the hottest days of the year.

MATERIALS AND METHODS

Our analytical approach has five key steps (fig. S1): (i) estimate the effect of extreme heat on growth using empirical regression methods at the subnational level; (ii) use climate model simulations to calculate counterfactual extreme heat time series for each region; (iii) use the empirical results along with the counterfactual heat wave data to calculate subnational economic growth changes due to anthropogenic changes in extreme heat; (iv) use country-level income data and regional nighttime luminosity data to generate continuous regional GDPpc time series; and (v) calculate regional income losses or benefits attributable to anthropogenic changes in extreme heat.

This approach allows us to estimate uncertainty introduced in each step of the analysis. When estimating individual parameters in the regression analysis and regional GDPpc prediction procedure, we bootstrap (uniform sampling with replacement) to estimate sampling uncertainty in the parameters. We also sample the residual uncertainty in regional GDPpc time series alongside parametric uncertainty in the statistical model used to predict regional GDPpc. We use multiple climate models, several with multiple realizations, to sample uncertainty in anthropogenic changes to extreme heat. Last, when calculating regional losses or benefits, we use a Monte Carlo approach that samples from each of these individual uncertainty distributions to propagate each source of uncertainty through the analysis. Details are provided in the individual sections below.

Data

Historical climate data come from the ERA5 reanalysis (*70*), from which we calculate annual mean temperature, temperature variability, the annual cycle, extreme heat (see the "Extreme temperature metric" section), and accumulated precipitation. We spatially average these data to the first subnational administrative level (or "region," such as states in the United States), weighting by population using year 2000 population data from the Gridded Population of the World (*71*). Population weighting is used to ensure that the climate data are aggregated in a way that reflects human exposure to climate hazards (*27*).

We merge our data with 1979–2016 regional GDPpc data assembled by Kalkuhl and Wenz (*31*) and provided by Kotz *et al.* (*33*). These data are a sample of opportunity derived primarily from national accounts data and yearbooks (*31*) and therefore reflect global inequities in institutional capacity, but they substantially advance our ability to track the subnational effects of climate extremes and are thus an important tool for our analysis. We correct for inflation by normalizing all data to 2010 price levels using the U.S. GDP deflator. Economic growth in a year is defined as the fractional difference in GDPpc relative to the previous year. The sample contains data from 1368 regions, each with between 4 and 38 years of data, for a total of 26,918 observations. Country-level GDPpc data are drawn from the World Bank World Development Indicators (*72*).

Climate models come from the CMIP6 (*55*). We use daily maximum temperature ("tasmax_day"), daily average temperature ("tas_day"), and monthly average temperature ("tas_Amon") data from the "historical," "historical-nat" (hereafter "natural"), and "ssp245" experiments (*56*, *73*) for all available realizations of eight models, for a total of 80 simulations (table S3). The natural experiments end in 2020, while the historical simulations end in 2014. Following the Detection and Attribution Model Intercomparison Project experimental protocol (*56*), we splice each historical simulation with the first 5 years of the corresponding ssp245 simulation to extend the historical simulations to 2020. This allows us to use a centered running mean through 2013 to smooth the data (see the Supplementary Materials). Model output is regridded to a common 1°-by-1° grid before analysis using bilinear interpolation from the "xarray" package in Python (*74*). Last, fossil fuel CO_2 emissions data come from the Community Emissions Data System (*75*), and year 2000 gridded crop yields for maize, wheat, rice, and soybeans come from EarthStat (*76*).

Extreme temperature metric

There are many ways to measure extreme heat, and a wide array of heat wave metrics has been proposed (*47*), each with benefits and drawbacks. Many studies calculate extreme heat relative to climatological baselines based on percentile-based thresholds that can vary on the basis of the location and time of year (*1*, *46*, *47*). However, while critical thresholds for extreme heat are well known for sectors such as agriculture in the United States (*22*, *66*), they are not necessarily known for aggregate economic production. Thus, while threshold-based extreme heat indices may be useful for future research in the economic impacts of climate change (*23*, *50*), the choice of thresholds is generally arbitrary.

Hence, we choose to represent extreme heat with a more objective measure: the average daily maximum temperature of the hottest

5-day period in each year, denoted "Tx5d." It is calculated by taking 5-day running means of daily maximum temperature at each grid cell over the 1979–2016 period, temporally aggregating to the annual maximum, and then spatially aggregating by calculating regional population-weighted averages. We focus on Tx5d because it is both simple and geophysically meaningful, measuring the hottest multiple-day period experienced in a year. It is also similar to metrics used in climate modeling studies of record-breaking temperatures (*1*, *11*, *44*). Calculating the maximum temperatures across a 5-day period also ensures that our analysis captures uniquely damaging multiday periods of extreme heat (*23*). Moreover, using Tx5d ensures that we measure temperatures during the hottest parts of the year, whereas seasonally varying thresholds would treat relatively warm periods during winter or shoulder seasons as equivalent to the hottest summer heat waves.

Our empirical identification strategy (see "Econometric analysis" section) interacts Tx5d with annual average temperature to incorporate heterogeneity in the response regions have to extreme heat. Different regions with different climatological baseline temperatures may respond differently to extreme heat. For example, warm regions may invest in technologies such as air conditioning due to their greater exposure to high temperatures (*9*). On the other hand, higher climatological temperatures mean that extreme heat may be more likely to cross physiological thresholds that are uniquely harmful to humans (*3*, *8*). Our analytical strategy therefore incorporates both an absolute metric of high temperature extremes and the relative ability of different populations in different regions to manage the risks of those high temperatures.

We calculate Tx5d values at grid cells and then average across regions. This strategy may average Tx5d values from different parts of the year within a single region. On the basis of analysis presented in detail in the Supplementary Material, this does not pose a problem for our analysis (fig. S13).

Last, although we focus on the simple and transparent Tx5d metric in our main analysis, we conduct a supplementary analysis where we recalculate extreme heat as deviations from location- and month-specific indices. This strategy allows us to simultaneously incorporate the intensity, frequency, and duration of extreme heat in one index, although comes with substantial costs in terms of complexity and arbitrary researcher choices. We find similar results as in our main analysis (see the Supplementary Materials), providing confidence that Tx5d is capturing the most impactful and damaging instances of extreme heat historically (fig. S14).

Econometric analysis

We use panel regression with fixed effects to model economic growth as a function of extreme high temperatures, temperature variability, and mean temperature, along with region-specific time-invariant characteristics and common global time-varying factors. This strategy separates extreme heat from other factors that might affect economic growth, allowing us to isolate the causal effect of heat intensity on the hottest days of the year. We estimate the following model for growth g in region i and year t with ordinary least squares

$$g_{it} = \alpha_1 T_{it} + \alpha_2 T_{it}^2 + \beta_1 Tx_{it} + \beta_2 Tx_{it^*} T_{it} + \gamma_1 V_{it} + \gamma_2 V_{it^*} A_i + \pi P_{it} + \mu_i + \delta_t + \epsilon_{it} \quad (1)$$

Here, T refers to annual mean temperature, Tx refers to Tx5d, V refers to temperature variability, A refers to the average annual cycle of temperature, and P refers to annual accumulated precipitation. Variability in each year is defined as the average within-month SD of daily mean temperatures, with the data drawn from Kotz *et al.* (*33*). The annual cycle is defined as the climatological average within-year difference of the maximum and minimum monthly temperature, following Kotz *et al.* (*33*). Kotz *et al.* (*33*) showed that regions with larger annual cycles (i.e., greater seasonality) are less harmed by temperature variability, since they may be acclimated to greater swings in temperature. Regions with modest annual cycles are also regions with high temperatures (i.e., the tropics), so the damages associated with both extremes and variability fall disproportionately on low-income tropical regions (fig. S5). Other terms include μ, which is a region fixed effect that controls for time-invariant regional differences such as geography, and δ, which is a year fixed effect that controls for common global shocks and global trends in extreme temperatures. The identifying assumption for the coefficients of interest is that after controlling for these spatial and temporal averages, Tx5d is plausibly exogenous with respect to the other factors that affect economic growth. Interacting Tx5d with annual average temperature allows the effect of extremes to vary on the basis of the underlying climate in a region. Last, our inclusion of average temperatures and temperature variability ensures that the effect of extremes we identify is independent of other parts of the temperature distribution (e.g., greater variability inducing more frequent crossing of extreme temperature thresholds).

To estimate sampling uncertainty in the regression coefficients, we estimate the parameters in Eq. 1 1000 times using a bootstrap resampling procedure, sampling with replacement from a uniform distribution of regions. Sampling by region—that is, keeping all years from a given region together to account for within-region autocorrelation in growth—is analogous to clustering standard errors by region (*28*). We also test parametric standard errors clustered by country, which accounts for simultaneous spatial and temporal correlation in growth, shown in table S1.

Year fixed effects may not sufficiently control for time-varying unobserved cofounders since these may be heterogeneous and not globally constant. Hence, we estimate an additional model where we add region-specific linear trends in growth to remove smoothly varying unobserved factors independently for each region, a common technique in the empirical climate-economy literature (*27*, *57*). The results from this regression yield even stronger effects for Tx5d, indicating that our main results should be viewed as conservative (table S1). Last, to examine whether the effects of extreme temperatures persist over time, we estimate a distributed lag version of the regression equation (see the Supplementary Materials).

Calculating regional growth changes from anthropogenic changes to extreme heat

To isolate the contribution of anthropogenic warming to extreme temperatures, we calculate population-weighted Tx5d at the regional scale from the CMIP6 historical and natural simulations. Counterfactual Tx5d values are calculated as the observed Tx5d time series minus the smoothed difference between the historical and natural simulations (see the Supplementary Materials).

We then apply the coefficients from the distributed lag regression model to the observed and counterfactual Tx5d time series. The difference between them represents the additional growth that would have occurred in the absence of human-induced changes in Tx5d. The result of this calculation is a region-scale time series of the change in economic growth, denoted Δg (eq. S6). Because this calculation only

requires climate data (i.e., the region's average temperature and Tx5d), we are able to calculate this growth difference for all regions, even where observed growth data are not available. The hatching in Fig. 1B shows where these data are not available.

Predicting continuous regional income time series

Because many regions in our dataset do not contain continuous GDPpc data, we develop a simple and parsimonious statistical model for predicting regional GDPpc data in areas where it is not currently available. We emphasize that we do not apply our empirical regression approach to this constructed data, but only use it in the calculation of accumulated economic damages from warming.

The predictand of interest is regional GDPpc in each year. The two key predictors we use are country-level GDPpc and regional average nighttime luminosity ("nightlights"). Country-level GDPpc is useful for predicting global variation in regional income; regions in high-income countries likely have higher incomes than regions in low-income countries. We find that country GDPpc alone explains some 87% of variation in regional GDPpc in our sample (because these data are in per capita terms, regional population does not appear to provide additional explanatory value). Nightlights are an additional useful predictor for explaining within-country variation in income; regions with higher luminosity at night are likely regions with more economic activity (*59*–*61*). All of the following analysis is performed over 1992–2013, which is the longest time period over which the nightlights data are available.

Our main statistical model predicts log regional GDPpc in country *c*, region *r*, and year *t* using country-level GDPpc, regional average nightlights *NL*, and their interaction. The interaction term is included because nightlights tend to saturate at high income levels, so their explanatory power lessens as income increases (*61*). We estimate the following regression model using ordinary least squares

$$\begin{aligned} \ln GDPpc_{ctr} = {} & \beta_0 + \beta_1 \ln GDPpc_{ct} + \beta_2 NL_{ctr} + \\ & \beta_3 \ln GDPpc_{ct} * NL_{ctr} + \epsilon_{ctr} \end{aligned} \quad (2)$$

All three parameters of interest (β_1, β_2, and β_3) are statistically significant ($P < 0.05$) and are shown in table S4. The predicted values from this equation are shown in fig. S8. Uzbekistan and Kenya appear to be outliers, potentially due to errors in the country-level GDPpc data (*77*). When these countries are included, the predicted data explain ~87% of variation in the observed data; when they are dropped, the predicted data explain ~90% of variation in the observed data (fig. S8).

To estimate and propagate sampling uncertainty in the parameters for this downscaling procedure, we bootstrap the Eq. 2 regression 1000 times, sampling from a uniform distribution of countries with replacement and using all years from a given country to preserve country-level autocorrelation in income. In addition, for each iteration, we add a realization of random noise from a Gaussian distribution with mean zero and variance equal to the variance of the residuals from the statistical model. The result is 1000 realizations of GDPpc for each region-year pair that sample both parametric and residual uncertainty in this downscaling procedure. These realizations are sampled as part of our wider Monte Carlo uncertainty analysis, discussed below.

We test the out-of-sample predictive power of this approach with a 10-fold cross-validation analysis. We split the data into 10 mutually exclusive training and testing datasets, splitting by country to preserve within-country correlations. We estimate the regression of interest for each training dataset and calculate the root mean squared prediction error for the testing dataset. The regressions tested include our main regression (Eq. 2) and alternative models with several sets of predictors: country-level GDPpc, regional yields of the four major crops (maize, wheat, rice, and soybeans) summed across crops and spatially averaged across regions, regional nightlights, regional area, and regional population. We test models that include each of these predictors alone, the combination of country-level GDPpc and each predictor independently, and a saturated model with all predictors. When nightlights and country-level GDPpc are included in the same model, they are interacted as described above.

All models that include country-level GDPpc have high out-of-sample predictive power, with prediction errors of less than 7% of the average regional log GDPpc (fig. S9). The model with the smallest out-of-sample prediction error includes only country-level GDPpc and regional nightlights, which is why we use it as our main prediction model. The low errors in this out-of-sample prediction procedure support our choice to infer GDPpc for regions where country-level data are available, but regional data are not.

To the best of our knowledge, our analysis is the first to produce a global sample of continuous GDPpc data at the regional level with an explicit treatment of parametric and residual uncertainty. Lessmann and Seidel (*61*) performed a similar analysis but did not use out-of-sample cross-validation to inform their choice of statistical model and did not generate multiple realizations to sample uncertainty. We make these data available to the community, independent of the replication data for the rest of our analysis, at the following location: github.com/ccallahan45/Global_Subnational_Income/

Damages from historical climate change

The climate model analysis yields a time series of the change in growth due to anthropogenic changes in Tx5d for each region (Δg). We then add this growth change to the downscaled GDPpc time series for each region and reintegrate each region's growth to calculate counterfactual GDPpc (*29*). The effect of anthropogenic changes in extreme temperatures is calculated as the difference between the observed and counterfactual time series.

Uncertainty in damages is calculated using a Monte Carlo analysis (N = 10,000) to incorporate and propagate uncertainty at each step of the causal chain. The individual steps of the analysis yield uncertainty distributions using multiple climate realizations or bootstrapping (in the case of the econometric regression and income downscaling). Each Monte Carlo iteration calculates damages after sampling, with replacement, one value from each of these distributions: one of the 80 climate model realizations, one of the 1000 bootstrapped econometric regression coefficients, and one of the 1000 regional GDPpc time series realizations. When selecting the climate model realizations, we adjust the sampling probabilities so that models with more realizations are reduced in probability, to make each model equally likely to be sampled. The other samples are from uniform distributions.

SUPPLEMENTARY MATERIALS

Supplementary material for this article is available at https://science.org/doi/10.1126/sciadv.add3726

REFERENCES AND NOTES

1. G. A. Meehl, C. Tebaldi, More intense, more frequent, and longer lasting heat waves in the 21st century. *Science* **305**, 994–997 (2004).
2. P. A. Stott, D. A. Stone, M. R. Allen, Human contribution to the European heatwave of 2003. *Nature* **432**, 610–614 (2004).

3. E. D. Coffel, R. M. Horton, A. de Sherbinin, Temperature and humidity based projections of a rapid rise in global heat stress exposure during the 21st century. *Environ. Res. Lett.* **13**, 014001 (2017).
4. A. M. Vicedo-Cabrera, N. Scovronick, F. Sera, D. Royé, R. Schneider, A. Tobias, C. Astrom, Y. Guo, Y. Honda, D. M. Hondula, R. Abrutzky, S. Tong, M. de S. Z. S. Coelho, P. H. N. Saldiva, E. Lavigne, P. M. Correa, N. V. Ortega, H. Kan, S. Osorio, J. Kyselý, A. Urban, H. Orru, E. Indermitte, J. J. K. Jaakkola, N. Ryti, M. Pascal, A. Schneider, K. Katsouyanni, E. Samoli, F. Mayvaneh, A. Entezari, P. Goodman, A. Zeka, P. Michelozzi, F. de'Donato, M. Hashizume, B. Alahmad, M. H. Diaz, C. D. L. C. Valencia, A. Overcenco, D. Houthuijs, C. Ameling, S. Rao, F. Di Ruscio, G. Carrasco-Escobar, X. Seposo, S. Silva, J. Madureira, I. H. Holobaca, S. Fratianni, F. Acquaotta, H. Kim, W. Lee, C. Iniguez, B. Forsberg, M. S. Ragettli, Y. L. L. Guo, B. Y. Chen, S. Li, B. Armstrong, A. Aleman, A. Zanobetti, J. Schwartz, T. N. Dang, D. V. Dung, N. Gillett, A. Haines, M. Mengel, V. Huber, A. Gasparrini, The burden of heat-related mortality attributable to recent human-induced climate change. *Nat. Clim. Change* **11**, 492–500 (2021).
5. L. J. Harrington, D. J. Frame, E. M. Fischer, E. Hawkins, M. Joshi, C. D. Jones, Poorest countries experience earlier anthropogenic emergence of daily temperature extremes. *Environ. Res. Lett.* **11**, 055007 (2016).
6. I. Mahlstein, R. Knutti, S. Solomon, R. W. Portmann, Early onset of significant local warming in low latitude countries. *Environ. Res. Lett.* **6**, 034009 (2011).
7. A. D. King, M. G. Donat, E. M. Fischer, E. Hawkins, L. V. Alexander, D. J. Karoly, A. J. Dittus, S. C. Lewis, S. E. Perkins, The timing of anthropogenic emergence in simulated climate extremes. *Environ. Res. Lett.* **10**, 094015 (2015).
8. S. C. Sherwood, M. Huber, An adaptability limit to climate change due to heat stress. *Proc. Natl. Acad. Sci. U.S.A.* **107**, 9552–9555 (2010).
9. T. A. Carleton, A. Jina, M. T. Delgado, M. Greenstone, T. Houser, S. M. Hsiang, A. Hultgren, R. E. Kopp, K. E. McCusker, I. B. Nath, "Valuing the global mortality consequences of climate change accounting for adaptation costs and benefits," Working paper, National Bureau of Economic Research, July 2020.
10. E. D. Coffel, R. M. Horton, J. M. Winter, J. S. Mankin, Nonlinear increases in extreme temperatures paradoxically dampen increases in extreme humid-heat. *Environ. Res. Lett.* **14**, 084003 (2019).
11. E. M. Fischer, S. Sippel, R. Knutti, Increasing probability of record-shattering climate extremes. *Nat. Clim. Change* **11**, 689–695 (2021).
12. S. Rahmstorf, D. Coumou, Increase of extreme events in a warming world. *Proc. Natl. Acad. Sci. U.S.A.* **108**, 17905–17909 (2011).
13. R. M. Horton, J. S. Mankin, C. Lesk, E. Coffel, C. Raymond, A review of recent advances in research on extreme heat events. *Curr. Clim. Change Rep.* **2**, 242–259 (2016).
14. D. J. Frame, L. J. Harrington, J. S. Fuglestvedt, R. J. Millar, M. M. Joshi, S. Caney, Emissions and emergence: A new index comparing relative contributions to climate change with relative climatic consequences. *Environ. Res. Lett.* **14**, 084009 (2019).
15. N. S. Diffenbaugh, Human well-being, the global emissions debt, and climate change commitment. *Sustain. Sci.* **8**, 135–141 (2013).
16. M. P. Byrne, Amplified warming of extreme temperatures over tropical land. *Nat. Geosci.* **14**, 837–841 (2021).
17. L. J. Harrington, F. E. Otto, Reconciling theory with the reality of African heatwaves. *Nat. Clim. Change.* **10**, 796–798 (2020).
18. F. E. L. Otto, L. Harrington, K. Schmitt, S. Philip, S. Kew, G. J. van Oldenborgh, R. Singh, J. Kimutai, P. Wolski, Challenges to understanding extreme weather changes in lower income countries. *Bull. Am. Meteorol. Soc.* **101**, E1851–E1860 (2020).
19. N. Stern, The structure of economic modeling of the potential impacts of climate change: Grafting gross underestimation of risk onto already narrow science models. *J. Econ. Lit.* **51**, 838–859 (2013).
20. M. Auffhammer, Quantifying economic damages from climate change. *J. Econ. Perspect.* **32**, 33–52 (2018).
21. E. Somanathan, R. Somanathan, A. Sudarshan, M. Tewari, The impact of temperature on productivity and labor supply: Evidence from Indian manufacturing. *J. Polit. Econ.* **129**, 1797–1827 (2021).
22. W. Schlenker, M. J. Roberts, Nonlinear temperature effects indicate severe damages to U.S. crop yields under climate change. *Proc. Natl. Acad. Sci. U.S.A.* **106**, 15594–15598 (2009).
23. S. Miller, K. Chua, J. Coggins, H. Mohtadi, Heat waves, climate change, and economic output. *J. Eur. Econ. Assoc.* **19**, 2658–2694 (2021).
24. S. M. Hsiang, Temperatures and cyclones strongly associated with economic production in the Caribbean and Central America. *Proc. Natl. Acad. Sci. U.S.A.* **107**, 15367–15372 (2010).
25. T. Deryugina, S. Hsiang, "The marginal product of climate," Working paper, National Bureau of Economic Research, November 2017.
26. D. García-León, A. Casanueva, G. Standardi, A. Burgstall, A. D. Flouris, L. Nybo, Current and projected regional economic impacts of heatwaves in Europe. *Nat. Commun.* **12**, 5807 (2021).
27. M. Burke, S. M. Hsiang, E. Miguel, Global non-linear effect of temperature on economic production. *Nature* **527**, 235–239 (2015).
28. M. Burke, W. M. Davis, N. S. Diffenbaugh, Large potential reduction in economic damages under UN mitigation targets. *Nature* **557**, 549–553 (2018).
29. N. S. Diffenbaugh, M. Burke, Global warming has increased global economic inequality. *Proc. Natl. Acad. Sci. U.S.A.* **116**, 9808–9813 (2019).
30. M. Burke, V. Tanutama, "Climatic constraints on aggregate economic output," Working paper, National Bureau of Economic Research, April 2019.
31. M. Kalkuhl, L. Wenz, The impact of climate conditions on economic production. Evidence from a global panel of regions. *J. Environ. Econ. Manag.* **103**, 102360 (2020).
32. M. Dell, B. F. Jones, B. A. Olken, Temperature shocks and economic growth: Evidence from the last half century. *Am. Econ. J. Macroecon.* **4**, 66–95 (2012).
33. M. Kotz, L. Wenz, A. Stechemesser, M. Kalkuhl, A. Levermann, Day-to-day temperature variability reduces economic growth. *Nat. Clim. Change* **11**, 319–325 (2021).
34. S. I. Seneviratne, N. Nicholls, D. Easterling, C. M. Goodess, S. Kanae, J. Kossin, Y. Luo, J. Marengo, K. McInnes, M. Rahimi, M. Reichstein, A. Sorteberg, C. Vera, X. Zhang, M. Rusticucci, V. Semenov, L. V. Alexander, S. Allen, G. Benito, T. Cavazos, J. Clague, D. Conway, P. M. Della-Marta, M. Gerber, S. Gong, B. N. Goswami, M. Hemer, C. Huggel, B. van den Hurk, V. V. Kharin, A. Kitoh, A. M. G. K. Tank, G. Li, S. Mason, W. McGuire, G. J. van Oldenborgh, B. Orlowsky, S. Smith, W. Thiaw, A. Velegrakis, P. Yiou, T. Zhang, T. Zhou, F. W. Zwiers, Changes in climate extremes and their impacts on the natural physical environment, in *Managing the Risks of Extreme Events and Disasters to Advance Climate Change Adaptation*, C. B. Field, V. Barros, T. F. Stocker, Q. Dahe, Eds. (Cambridge Univ. Press, 2012), pp. 109–230.
35. S. I. Seneviratne, M. G. Donat, B. Mueller, L. V. Alexander, No pause in the increase of hot temperature extremes. *Nat. Clim. Change* **4**, 161–163 (2014).
36. D. E. Horton, N. C. Johnson, D. Singh, D. L. Swain, B. Rajaratnam, N. S. Diffenbaugh, Contribution of changes in atmospheric circulation patterns to extreme temperature trends. *Nature* **522**, 465–469 (2015).
37. E. Black, M. Blackburn, G. Harrison, B. Hoskins, J. Methven, Factors contributing to the summer 2003 European heatwave. *Weather* **59**, 217–223 (2004).
38. E. M. Fischer, S. I. Seneviratne, D. Lüthi, C. Schär, Contribution of land-atmosphere coupling to recent European summer heat waves. *Geophys. Res. Lett.* **34**, L06707 (2007).
39. D. G. Miralles, A. J. Teuling, C. C. Van Heerwaarden, J. V.-G. de Arellano, Mega-heatwave temperatures due to combined soil desiccation and atmospheric heat accumulation. *Nat. Geosci.* **7**, 345–349 (2014).
40. M. M. Vogel, R. Orth, F. Cheruy, S. Hagemann, R. Lorenz, B. J. van den Hurk, S. I. Seneviratne, Regional amplification of projected changes in extreme temperatures strongly controlled by soil moisture-temperature feedbacks. *Geophys. Res. Lett.* **44**, 1511–1519 (2017).
41. M. G. Donat, A. J. Pitman, S. I. Seneviratne, Regional warming of hot extremes accelerated by surface energy fluxes. *Geophys. Res. Lett.* **44**, 7011–7019 (2017).
42. B. Lyon, A. G. Barnston, E. Coffel, R. M. Horton, Projected increase in the spatial extent of contiguous US summer heat waves and associated attributes. *Environ. Res. Lett.* **14**, 114029 (2019).
43. C. Gessner, E. M. Fischer, U. Beyerle, R. Knutti, Very rare heat extremes: Quantifying and understanding using ensemble reinitialization. *J. Climate* **34**, 6619–6634 (2021).
44. N. S. Diffenbaugh, F. V. Davenport, On the impossibility of extreme event thresholds in the absence of global warming. *Environ. Res. Lett.* **16**, 115014 (2021).
45. D. Barriopedro, E. M. Fischer, J. Luterbacher, R. M. Trigo, R. García-Herrera, The hot summer of 2010: Redrawing the temperature record map of Europe. *Science* **332**, 220–224 (2011).
46. J. Sillmann, V. V. Kharin, X. Zhang, F. W. Zwiers, D. Bronaugh, Climate extremes indices in the CMIP5 multimodel ensemble: Part 1. Model evaluation in the present climate. *J. Geophys. Res. Atmospheres* **118**, 1716–1733 (2013).
47. S. E. Perkins, L. V. Alexander, On the measurement of heat waves. *J. Climate* **26**, 4500–4517 (2013).
48. R. G. Newell, B. C. Prest, S. E. Sexton, The GDP-temperature relationship: Implications for climate change damages. *J. Environ. Econ. Manag.* **108**, 102445 (2021).
49. A. Barreca, K. Clay, O. Deschenes, M. Greenstone, J. S. Shapiro, Adapting to climate change: The remarkable decline in the US temperature-mortality relationship over the twentieth century. *J. Polit. Econ.* **124**, 105–159 (2016).
50. M. Kotz, A. Levermann, L. Wenz, The effect of rainfall changes on economic production. *Nature* **601**, 223–227 (2022).
51. L. Berrang-Ford, A. R. Siders, A. Lesnikowski, A. P. Fischer, M. W. Callaghan, N. R. Haddaway, K. J. Mach, M. Araos, M. A. R. Shah, M. Wannewitz, D. Doshi, T. Leiter, C. Matavel, J. I. Musah-Surugu, G. Wong-Parodi, P. Antwi-Agyei, I. Ajibade, N. Chauhan, W. Kakenmaster, C. Grady, V. I. Chalastani, K. Jagannathan, E. K. Galappaththi, A. Sitati, G. Scarpa, E. Totin, K. Davis, N. C. Hamilton, C. J. Kirchhoff, P. Kumar, B. Pentz, N. P. Simpson, E. Theokritoff, D. Deryng, D. Reckien, C. Zavaleta-Cortijo, N. Ulibarri, A. C. Segnon, V. Khavhagali, Y. Shang, L. Zvobgo, Z. Zommers, J. Xu, P. A. Williams, I. V. Canosa, N. van Maanen, B. van Bavel, M. van Aalst, L. L. Turek-Hankins, H. Trivedi,

C. H. Trisos, A. Thomas, S. Thakur, S. Templeman, L. C. Stringer, G. Sotnik, K. D. Sjostrom, C. Singh, M. Z. Siña, R. Shukla, J. Sardans, E. A. Salubi, L. S. S. Chalkasra, R. Ruiz-Díaz, C. Richards, P. Pokharel, J. Petzold, J. Penuelas, J. P. Avila, J. B. P. Murillo, S. Ouni, J. Niemann, M. Nielsen, M. New, P. N. Schwerdtle, G. N. Alverio, C. A. Mullin, J. Mullenite, A. Mosurska, M. D. Morecroft, J. C. Minx, G. Maskell, A. M. Nunbogu, A. K. Magnan, S. Lwasa, M. Lukas-Sithole, T. Lissner, O. Lilford, S. F. Koller, M. Jurjonas, E. T. Joe, L. T. M. Huynh, A. Hill, R. R. Hernandez, G. Hegde, T. Hawxwell, S. Harper, A. Harden, M. Haasnoot, E. A. Gilmore, L. Gichuki, A. Gatt, M. Garschagen, J. D. Ford, A. Forbes, A. D. Farrell, C. A. F. Enquist, S. Elliott, E. Duncan, E. C. de Perez, S. Coggins, T. Chen, D. Campbell, K. E. Browne, K. J. Bowen, R. Biesbroek, I. D. Bhatt, R. B. Kerr, S. L. Barr, E. Baker, S. E. Austin, I. Arotoma-Rojas, C. Anderson, W. Ajaz, T. Agrawal, T. Z. Abu, A systematic global stocktake of evidence on human adaptation to climate change. *Nat. Clim. Change* **11**, 989–1000 (2021).

52. L. L. Turek-Hankins, E. Coughlan de Perez, G. Scarpa, R. Ruiz-Diaz, P. N. Schwerdtle, E. T. Joe, E. K. Galappaththi, E. M. French, S. E. Austin, C. Singh, M. Siña, A. R. Siders, M. K. van Aalst, S. Templeman, A. M. Nunbogu, L. Berrang-Ford, T. Agrawal; the Global Adaptation Mapping Initiative team, K. J. Mach, Climate change adaptation to extreme heat: A global systematic review of implemented action. *Oxf. Open Clim. Change* **1**, kgab005 (2021).
53. T. W. Ruff, J. D. Neelin, Long tails in regional surface temperature probability distributions with implications for extremes under global warming. *Geophys. Res. Lett.* **39**, L04704 (2012).
54. P. Huybers, K. A. McKinnon, A. Rhines, M. Tingley, U.S. daily temperatures: The meaning of extremes in the context of nonnormality. *J. Climate* **27**, 7368–7384 (2014).
55. V. Eyring, S. Bony, G. A. Meehl, C. A. Senior, B. Stevens, R. J. Stouffer, K. E. Taylor, Overview of the Coupled Model Intercomparison Project phase 6 (CMIP6) experimental design and organization. *Geosci. Model Dev.* **9**, 1937–1958 (2016).
56. N. Gillett, H. Shiogama, B. Funke, G. Hegerl, R. Knutti, K. Matthes, B. Santer, D. Stone, C. Tebaldi, The detection and attribution model intercomparison project (DAMIP v1. 0) contribution to CMIP6. *Geosci. Model Dev.* **9**, 3685–3697 (2016).
57. S. M. Hsiang, A. S. Jina, "The causal effect of environmental catastrophe on long-run economic growth: Evidence from 6,700 cyclones," Working paper, National Bureau of Economic Research, July 2014.
58. F. C. Moore, D. B. Diaz, Temperature impacts on economic growth warrant stringent mitigation policy. *Nat. Clim. Change.* **5**, 127–131 (2015).
59. X. Chen, W. D. Nordhaus, Using luminosity data as a proxy for economic statistics. *Proc. Natl. Acad. Sci. U.S.A.* **108**, 8589–8594 (2011).
60. J. V. Henderson, A. Storeygard, D. N. Weil, Measuring economic growth from outer space. *Am. Econ. Rev.* **102**, 994–1028 (2012).
61. C. Lessmann, A. Seidel, Regional inequality, convergence, and its determinants – A view from outer space. *Eur. Econ. Rev.* **92**, 110–132 (2017).
62. M. D. Mastrandrea, C. B. Field, T. F. Stocker, O. Edenhofer, K. L. Ebi, D. J. Frame, H. Held, E. Kriegler, K. J. Mach, P. R. Matschoss, G.-K. Plattner, G. W. Yohe, F. W. Zwiers, *Guidance Note for Lead Authors of the IPCC Fifth Assessment Report on Consistent Treatment of Uncertainties* (Intergovernmental Panel on Climate Change, 2010).
63. S. I. Seneviratne, M. G. Donat, A. J. Pitman, R. Knutti, R. L. Wilby, Allowable CO_2 emissions based on regional and impact-related climate targets. *Nature* **529**, 477–483 (2016).
64. C. W. Callahan, J. S. Mankin, National attribution of historical climate damages. *Clim. Change* **172**, 40 (2022).
65. M. Kotz, L. Wenz, A. Levermann, Footprint of greenhouse forcing in daily temperature variability. *Proc. Natl. Acad. Sci. U.S.A.* **118**, e2103294118 (2021).
66. E. E. Butler, P. Huybers, Adaptation of US maize to temperature variations. *Nat. Clim. Change* **3**, 68–72 (2013).
67. M. Burke, A. Driscoll, D. B. Lobell, S. Ermon, Using satellite imagery to understand and promote sustainable development. *Science* **371**, eabe8628 (2021).
68. E. D. Coffel, A. de Sherbinin, R. M. Horton, K. Lane, S. Kienberger, O. Wilhelmi, Chapter 7 - The science of adaptation to extreme heat, in *Resilience*, Z. Zommers, K. Alverson, Eds. (Elsevier, 2018), pp. 89–103.
69. M. Pascal, K. Laaidi, M. Ledrans, E. Baffert, C. Caserio-Schönemann, A. Le Tertre, J. Manach, S. Medina, J. Rudant, P. Empereur-Bissonnet, France's heat health watch warning system. *Int. J. Biometeorol.* **50**, 144–153 (2006).
70. C3S, ERA5: Fifth generation of ECMWF atmospheric reanalysis of the global climate. *Copernic. Clim. Change Serv. Clim. Data Store* **15**, 2022 (2017).
71. C. U. Center for International Earth Science Information Network CIESIN, *Gridded Population of the World, Version 4 (GPWv4): Population Count* (2016).
72. T. W. Bank, *World Development Indicators 2016* (2016).
73. B. C. O'Neill, C. Tebaldi, D. P. van Vuuren, V. Eyring, P. Friedlingstein, G. Hurtt, R. Knutti, E. Kriegler, J.-F. Lamarque, J. Lowe, G. A. Meehl, R. Moss, K. Riahi, B. M. Sanderson, The scenario model intercomparison project (ScenarioMIP) for CMIP6. *Geosci. Model Dev.* **9**, 3461–3482 (2016).
74. S. Hoyer, J. Hamman, xarray: N-D labeled arrays and datasets in Python. *J Open Res Softw. Revis.* **5**, 10 (2017).
75. R. M. Hoesly, S. J. Smith, L. Feng, Z. Klimont, G. Janssens-Maenhout, T. Pitkanen, J. J. Seibert, L. Vu, R. J. Andres, R. M. Bolt, T. C. Bond, L. Dawidowski, N. Kholod, J.-i. Kurokawa, M. Li, L. Liu, Z. Lu, M. C. P. Moura, P. R. O'Rourke, Q. Zhang, Historical (1750–2014) anthropogenic emissions of reactive gases and aerosols from the Community Emission Data System (CEDS). *Geosci. Model Dev.* **11**, 369–408 (2018).
76. C. Monfreda, N. Ramankutty, J. A. Foley, Farming the planet: 2. Geographic distribution of crop areas, yields, physiological types, and net primary production in the year 2000. *Glob. Biogeochem. Cycles.* **22**, GB1022 (2008).
77. A. Deaton, A. Heston, Understanding PPPs and PPP-based national accounts. *Am. Econ. J. Macroecon.* **2**, 1–35 (2010).

Acknowledgments: We thank Dartmouth's Research Computing and the Discovery Cluster for computing resources and the World Climate Research Programme, which, through its Working Group on Coupled Modeling, coordinated and promoted CMIP6. **Funding:** This work was supported by NSF Graduate Research Fellowship #1840344 to C.W.C. and grants from Dartmouth's Wright Center for the Study of Computation and Just Communities, Dartmouth's Neukom Computational Institute, and the Nelson A. Rockefeller Center to J.S.M. **Author contributions:** Both authors conceived the study and designed the analysis. C.W.C. performed the analysis. Both authors interpreted the results and wrote the manuscript. **Competing interests:** The authors declare that they have no competing interests. **Data and materials availability:** All data needed to evaluate the conclusions in the paper are present in the paper and/or the Supplementary Materials. Processed data and analysis code that support the findings of this study are available at github.com/ccallahan45/CallahanMankin_ExtremeHeatEconomics_2022/, permanently archived on Zenodo at doi.org/10.5281/zenodo.7017229. The ERA5 reanalysis data are publicly available at www.ecmwf.int/en/forecasts/datasets/reanalysis-datasets/era5, the CMIP6 climate model data are publicly available at https://esgf-node.llnl.gov/search/cmip6/, GADM shapefile data are publicly available at https://gadm.org/download_world.html, and Palmer Drought Severity Index data are publicly available at https://crudata.uea.ac.uk/cru/data/drought/.

Submitted 7 June 2022
Accepted 9 September 2022
Published 28 October 2022
10.1126/sciadv.add3726
