# Supplementary material for: Globally unequal effect of extreme heat on economic growth
Source: Sci Adv. 2022 Oct 28;8(43):eadd3726. doi: 10.1126/sciadv.add3726 (PMC9616493; doi:10.1126/sciadv.add3726)
Supplement: Supplementary file 3 — Erratum published 27 October 2023: The authors discovered a code error underlying Fig. 4, which does not change the conclusions of the paper. However, the correction to the code did change some values reported in the Abstract, the Results, Fig. 4, and figure S11. The PDF, HTML, Supplementary Materials, and Fig. 4 have been updated. The original version of the Supplementary Materials is available here: [file sciadv.add3726_sm.v1.pdf]

Science Advances
AAAS

Supplementary Materials for

Globally unequal effect of extreme heat on economic growth

Christopher W. Callahan and Justin S. Mankin

Corresponding author: Christopher W. Callahan, christopher.w.callahan.gr@dartmouth.edu

Sci. Adv. **8**, eadd3726 (2022)
DOI: 10.1126/sciadv.add3726

This PDF file includes:

Supplementary Methods
Tables S1 to S4
Figs. S1 to S14

Supplementary Methods

Here we show: (1) the results of a placebo test to ensure that spurious trends are not driving our result; (2) details on how we calculate and extrapolate marginal effects of extreme temperatures; (3) details on how we estimate a distributed lag version of our regression analysis; (4) details on how we calculate regional growth changes from anthropogenic changes in extreme heat; (5) discussion of the spatial aggregation of Tx5d to subnational regions; and (6) an exploration of an alternative methodology using a threshold-based index of extreme heat.

Placebo test

To ensure that our primary regression results do not arise by chance, we implement several placebo tests. We do this by re-calculating the regression coefficients after repeatedly (N = 1000) randomizing Tx5d exposure using three different schemes: (1) across the entire sample; (2) within individual years; and (3) within individual regions. The results from these three randomization schemes are presented in Fig. S2. If spurious trends across time or space were driving our results, these tests would yield significant coefficients (as in our original model) despite randomizing extreme heat exposure with respect to growth. However, the results of these tests support our results and the soundness of our identification strategy: as expected, when randomizing the treatment, the effect of extreme heat on growth disappears (Fig. S2). These results provide confidence that spurious trends or a flawed identification strategy are not driving our results.

Calculating and extrapolating marginal effects

Using the main regression equation (main text Eq. 1), we differentiate *g* with respect to each variable. This allows us to calculate marginal effects of each variable on growth. The marginal growth effect of an additional degree in Tx5d (p.p. per °C) in a given region (i.e., $\frac{\partial g_{it}}{\partial Tx_{it}}$) depends on that region's annual average temperature:

$$\frac{\partial g_{it}}{\partial Tx_{it}} = \beta_1 + \beta_2 * T_{it} \tag{1}$$

The marginal effect of an additional degree of temperature variability (*V*, p.p. per °C) in a given region depends on the annual cycle, *A*:

$$\frac{\partial g_{it}}{\partial V_{it}} = \gamma_1 + \gamma_2 * A_i \quad (2)$$

The annual mean temperature term (*T*) is interacted both with itself (i.e, T^2) and with Tx5d. The squared temperature term is not statistically significant (Table S1), so we calculate the marginal effect of an additional degree in the annual mean temperature (p.p. per °C) using only the interaction with Tx5d:

$$\frac{\partial g_{it}}{\partial T_{it}} = \alpha_1 + \beta_2 Tx_{it} \quad (3)$$

We present the marginal effects of extreme heat, annual mean temperature, and temperature variability in Supplementary Figure 5. We standardize all marginal effects by their respective within-region standard deviations. For example, the average region experiences variability in Tx5d of approximately 1.3 °C from year to year, so the effect of realistic variation in Tx5d is 1.3 times larger than would be inferred from the marginal effect of a 1 °C change. And because a 1 °C change has a different physical interpretation if it is in extreme heat, average temperatures, or temperature variability, standardizing the marginal effects of each variable allows us to make fair comparisons between them (Fig. S5).

Not all regions have GDP per capita (GDPpc) data; the estimation sample does not include most regions in Africa, some regions in South America, and other missing areas. However, climate data is available for these regions. To estimate the marginal effects for all regions, we calculate each region's effects based on its long-term average temperature. The primary regions of extrapolation are in the tropics (e.g., sub-Saharan Africa), which are areas of high temperatures. The estimation sample includes annual mean temperatures as high as 30 °C, so most of the regions for which we calculate marginal effects are within the range covered by the estimation sample.

Distributed lag model estimation

To determine whether the effects of extreme temperatures persist beyond the year in which they occur, we estimate a distributed lag version of our main regression specification. This model adds lags to each independent variable to account for potential economic recovery behavior after an extreme heat event. If climate shocks fall only on the *level* of output, the year after an event will see *increased* growth as economies rebound to their pre-heat-wave income trajectory, but if climate shocks affect *growth*, then future years will not rebound. Hence, if the lagged terms in a regression have the opposite sign of the contemporaneous effect, the cumulative effect will converge to zero, characteristic of level effects. On the other hand, if the lagged terms are zero or have the same sign as the contemporaneous effect, growth effects are identified.

The distributed lag model is specified as follows, where the cumulative effect is the original model summed across the number of lags L considered:

$$g_{it} = \sum_{L=0}^{j} \left[\alpha_{L1} T_{i(t-L)} + \alpha_{L2} T_{i(t-L)}^2 + \beta_{L1} Tx_{i(t-L)} + \beta_{L2} Tx_{i(t-L)} * T_{i(t-L)} + \gamma_{L1} V_{i(t-L)} + \gamma_{L2} V_{i(t-L)} * A_i + \pi_L P_{i(t-L)} \right] + \mu_i + \delta_t + \epsilon_{it} \quad (4)$$

As in Eqn. 1 (main text), T represents annual mean temperature, Tx represents Tx5d, V represents daily-scale variability, A represents the annual cycle, and P represents precipitation. The coefficient of interest shown in Fig. 3 in the main is the sum of the coefficients from lags L=0 to L=j:

$$\Theta_{ij} = \sum_{L=0}^{j} \left[\beta_{L1} + \beta_{L2} * T_{i(t-L)} \right] \quad (5)$$

Because each region has a unique temperature, each region has a unique cumulative marginal effect. In our analysis, we set j=5, meaning we add 5 lags (i.e., years) to the contemporaneous effect and estimate the effect of a heat wave on each year for 6 years in total. In Fig. 3, we show the cumulative coefficients for two example temperatures as the effect is accumulated over the 6 years. Other analyses occasionally include up to 15 or 20 lags, but because some regions in our sample have 10 or fewer years of data, we only include 5 lags in our model to avoid overfitting. This choice is defensible, as the cumulative marginal effects rebound to zero within two to three years of the extreme heat event (Fig. 3A).

Calculating regional growth changes from anthropogenic changes to extreme heat

Combining our empirical analysis with differences in the CMIP6 historical and natural simulations (Table S3) allows us to calculate the effect of anthropogenic changes in extreme heat on regional growth and income over the historical period. We first splice each model's historical simulation, which ends in 2014, with the first five years of its ssp245 simulation, extending the simulations to 2020 to match the natural simulations, which end in 2020. Our choice of the ssp245 scenario follows the CMIP6 experimental protocol.

We then calculate the difference in annual population-weighted region-mean Tx5d between the historical and natural simulations, and we smooth this difference with a centered fifteen-year running mean to isolate the secular effect of anthropogenic warming. We also perform this analysis for the annual mean temperature in each model and region. We use an analysis period of 1992-2013 to match the availability of economic and nighttime luminosity data, so we begin with climate model data over 1985-2020. The fifteen-year centered smoothing means that the value for 2010, for example, in the resulting 1992-2013 climate model timeseries represents the average from 2003 to 2017. Using the unsmoothed difference may result in spurious differences on interannual timescales from, for example, random El

Niño events that occur in one simulation, but not another, that are not attributable to anthropogenic forcing.

Because the natural simulations end in 2020—and there is no ssp245-type simulation that could be used to extend the natural simulations—fifteen years is the longest smoothing window that allows our estimates to use a centered window rather than a right-oriented window. Using a longer period may be useful for eliminating multidecadal natural climate variability, but it comes at the sacrifice of robustly capturing anthropogenic warming, since using a right-oriented window requires averaging years earlier than the year in question but not later than that year.

We then subtract this difference from each region's observed Tx5d time series (Tx^O) to generate counterfactual Tx5d timeseries (Tx^{CF}) that would have occurred absent anthropogenic greenhouse gas and aerosol emissions. Benchmarking the counterfactual timeseries to the observed time series both implicitly bias-corrects the model output and imputes realistic interannual variability to the smoothed climate model timeseries. We perform the same subtraction for annual mean temperature as well, yielding time series of observed (T^O) and counterfactual (T^{CF}) annual mean temperature.

We then compare the growth effects of Tx5d in the observed and counterfactual climates to calculate the difference in growth due to anthropogenic changes in extreme heat. We use the coefficients from the distributed lag model (Eqn. 4) in this procedure, allowing us to capture the full dynamics of the effect over time. The coefficients are derived in Eqn. 4 by estimating the effect of past extreme heat on current growth but are mathematically equivalent to the effect of current extreme heat on future growth. That is, β_3 is estimated as the effect of extreme heat at time *t-3* on growth at time *t*, which is equivalent to the effect of extreme heat at time *t* on growth at time *t+3*. This equivalency allows us to develop impulse-response functions that describe the response of future growth to a change in extreme heat at a given time (e.g., Fig. 3). Therefore, if anthropogenic forcing has altered Tx5d in year *t* and region *i*, the effect of that change on growth from time *t* to time *t+L* is calculated as:

$$\Delta g_{t+L} = [\beta_{L1} Tx_t^{CF} + \beta_{L2} Tx_t^{CF} * T_t^{CF}] - [\beta_{L1} Tx_t^{O} + \beta_{L2} Tx_t^{O} * T_t^{O}] \quad (6)$$

This analytical structure allows changes in Tx5d to affect both contemporaneous and future growth, incorporating the intensification and rebound effects seen in Figure 3A in the main. We use the β coefficients from the distributed lag model, with one modification: we force the coefficients to sum to zero in year 3, given that the effects in year 3 are indistinguishable from zero (Fig. 3). This yields a Δg value that accumulates over years 0, 1, and 2, and then converges to 0 in year 3. We note that our methodology yields positive Δg values for regions harmed by warming, as they would have grown faster in the absence of warming (though we flip the sign of Δg in our presentation in Fig. 3, so negative values indicate damage from warming, for visualization purposes).

We repeat this analysis for each year, yielding timeseries of Δg values where each value is the combined influence of the Tx5d effects from that year and the three years before it. We perform this calculation for all regions, since Tx5d and temperature data are available even where GDPpc data is not. We perform this analysis over 1992-2013, as opposed to the entire sample period, to overlap with the period of the nightlights data used to infer regional GDPpc (Methods).

Note that we use both observed and counterfactual annual mean temperature in this calculation (T^O and T^{CF}). As a result, our damages calculations incorporate both changes in Tx5d values themselves as well as changes in the sensitivity of growth to Tx5d (Fig. 1). Fig. S10 shows a version of our analysis where historical average temperatures are held at their observed values.

Spatial aggregation of Tx5d

We calculate Tx5d values at each grid cell before averaging across regions. This procedure may average Tx5d values from different parts of the year if, for example, one grid cell in a region experiences their hottest heat wave in June and another experiences their hottest heat wave in August. However, this mismatch should not affect our results. Just as GDP aggregates across multiple sectors and time periods to generate a summary measure of economic activity, aggregating across multiple events or time periods is a standard procedure to calculate summary measures of exposure to climate hazards. Our results should not be interpreted as measuring the impact of a specific heat wave in each region, but instead as the temperature of the hottest heat wave experienced by the average grid cell in each region.

To explore the issue of spatial aggregation further, we examine the day of year of the hottest five-day period at each grid cell (Fig. S13). The timing of the Tx5d event varies across large spatial scales rather than fine scales; for example, the northern part of sub-Saharan Africa generally experiences it in February and March, at the end of Southern Hemisphere summer, as the Intertropical Convergence Zone has moved south and dry conditions prevail north of the equator (Fig. S13A). We quantify this spatial variation by calculating the within-region standard deviation in Tx5d timing for all regions in the Northern Hemisphere (Fig. S13B). (Before performing this calculation, we shift the year in the Southern Hemisphere so day 0 corresponds to July 1 and day 365 corresponds to June 30 of the following year.) Regions generally experience variation in Tx5d timing of 1-2 weeks, with 74% of regions experiencing spatial variation of less than 14 days. This time scale is consistent with the spatial and temporal scales of the synoptic circulation anomalies that drive heat waves.

Effect of extreme degree days on economic growth

Our main analysis uses Tx5d because it is a simple and transparent metric with a straightforward physical interpretation. Alternative approaches to quantifying extreme heat use percentile-based metrics

that allow researchers to quantify both the magnitude and frequency of extreme heat exposure. Here we conduct preliminary analysis using a similar metric to test the sensitivity of our results to this choice.

We define an "extreme degree days" (EDDs) metric as accumulated exposure to temperatures above thresholds defined as location- and month-specific temperature thresholds. Using 1979-2016 as the reference period, we calculate the 95th percentile temperature value separately for each month and each grid cell in the ERA5 daily maximum temperature (Tx) data. We then calculate the difference of Tx on each day of the year and grid point from this 95th-percentile temperature threshold, setting negative differences to zero. Annual EDDs are then calculated as the sum of these Tx differences over each year, limiting the summation to threshold exceedances of at least three consecutive days. This choice ensures that our analysis measures damaging multi-day periods of extreme temperatures.

We then substitute this EDDs metric for the Tx5d metric in our main regression equation (main text Eqn. 1) and re-estimate the regression with bootstrapping, as in our main analysis. The results are similar to those using Tx5d (Fig. S14): Extreme heat benefits cool regions and damages warm ones. The magnitude of the effect is similar, though slightly smaller, to Tx5d. In Brazil, where the average region has an annual mean temperature of 23.8 °C, a 1-s.d. increase in Tx5d intensity decreases growth by 0.63 p.p., whereas a 1-s.d. increase in EDDs decreases growth by 0.6 p.p. The similarity in magnitudes of the two effects suggests that the majority of the effect of extreme heat is felt during the few warmest days of the year (i.e., those days captured by Tx5d).

More broadly, these results provide confidence that percentile-based indices return similar results as simpler indices such as Tx5d. However, they require a series of arbitrary choices by researchers: Which percentile or absolute threshold to use, which time period to reference such a threshold to, which seasons to aggregate over, whether regions "recover" between periods of extreme heat within the same year, and whether the threshold changes with warming. The combined effect of these choices is that, while useful in capturing both the frequency and intensity of extreme heat, metrics such as EDDs can be complex to interpret and sensitive to the methodological choices made. We choose to maintain the simpler and more transparent Tx5d metric in our analysis while noting that threshold-based indices may be promising areas for future work. Additionally, because global warming will likely increase the frequency, magnitude, and duration of heat waves, incorporating these additional factors would likely produce more severe damages due to anthropogenic warming, so our main estimates can be viewed as conservative.

Supplementary Tables and Figures

	Dependent variable: growth (p.p.)			
	(1)	(2)	(3)	(4)
Tx5d	0.8689***	0.8694***	0.8689*	0.9460***
	(0.1483)	(0.1482)	(0.3489)	(0.1565)
Tx5d × T	−0.0627***	−0.0626***	−0.0627*	−0.0677***
	(0.0139)	(0.0139)	(0.0280)	(0.0148)
T	1.1503**	1.1467**	1.1503	1.3584**
	(0.3875)	(0.3892)	(0.6425)	(0.4158)
T^2	−0.0125	−0.0125	−0.0125	−0.0143
	(0.0152)	(0.0152)	(0.0395)	(0.0163)
Variability	−10.9322***	−10.9343***	−10.9322**	−11.5475***
	(1.5624)	(1.5627)	(3.6445)	(1.6585)
Variability × ann. cycle	0.2024**	0.2025**	0.2024	0.2142**
	(0.0634)	(0.0634)	(0.1524)	(0.0670)
Precipitation	0.5158	0.6022	0.5158	1.0163
	(0.4672)	(0.6896)	(1.4234)	(0.5310)
Precipitation^2		−0.0118		
		(0.0359)		
Observations	26918	26918	26918	26918
R^2	0.2107	0.2107	0.2107	0.2746
Adjusted R^2	0.1678	0.1678	0.1678	0.1928
Clustering	Region	Region	Country	Region
Trends	No	No	No	Yes

$^{***}p < 0.001$; $^{**}p < 0.01$; $^{*}p < 0.05$

Supplementary Table 1: Effects of extreme heat, average annual temperature, temperature variability, and precipitation on economic growth in percentage points (p.p.). Year and region fixed effects are included in all models. Original coefficients are multiplied by 100 to aid readability, so they are directly interpretable as percentage points. Column (1) shows our preferred model, column (2) adds a squared precipitation term, column (3) clusters standard errors at the country level instead of the regional level, and column (4) adds region-specific linear growth trends.

	Dependent variable: growth (p.p.)					
	(1)	(2)	(3)	(4)	(5)	(6)
T	1.1503**	−0.5100	−0.0531	1.2587***	−0.0304	1.1600**
	(0.3875)	(0.3042)	(0.2827)	(0.3528)	(0.2915)	(0.3885)
T^2	−0.0125	−0.0240	−0.0470***	−0.0118	−0.0454**	−0.0113
	(0.0152)	(0.0141)	(0.0133)	(0.0154)	(0.0138)	(0.0151)
Tx5d	0.8689***	0.8896***	0.1862	1.6126*	0.2271**	0.8870***
	(0.1483)	(0.1481)	(0.7948)	(0.8071)	(0.0752)	(0.1501)
Tx5d × T	−0.0627***			−0.0671***		−0.0622***
	(0.0139)			(0.0135)		(0.0138)
Tx5d × $\overline{\text{T}}$		−0.0657***				
		(0.0143)				
Tx5d × $\overline{\text{logGDPpc}}$			0.0016	−0.0781		
			(0.0842)	(0.0831)		
Tx5d × PDSI					0.0035	0.0031
					(0.0023)	(0.0023)
Observations	26918	26918	26918	26918	26918	26918
R^2	0.2107	0.2107	0.2100	0.2107	0.2100	0.2107
Adjusted R^2	0.1678	0.1679	0.1671	0.1678	0.1672	0.1679

$^{***}p < 0.001$; $^{**}p < 0.01$; $^{*}p < 0.05$

Supplementary Table 2: Effect of extreme heat on economic growth using different measures of effect heterogeneity. T refers to annual average temperature and T-bar refers to long-term climatological average temperature. PDSI refers to the Palmer Drought Severity Index. Precipitation, temperature variability, and the interaction between variability and the annual cycle are included in all models but not shown. Region and year fixed effects are included in all models. All standard errors clustered at the region level. Original coefficients are multiplied by 100 to aid readability, so they are directly interpretable as percentage points.

Model	Modeling center	Realizations
ACCESS-CM2	Commonwealth Scientific and Industrial Research Organization	3
ACCESS-ESM1-5	Commonwealth Scientific and Industrial Research Organization	3
CanESM5	Canadian Centre for Climate Modelling and Analysis	10
CNRM-CM6-1	Centre National de Recherches Meteorologiques/Centre Europeen de Recherche et Formation Avancee en Calcul Scientifique	1
FGOALS-g3	State Key Laboratory for Numerical Modeling for Atmospheric Science and Geophysical Fluid Dynamics	1
IPSL-CM6A-LR	Institut Pierre-Simon Laplace	5
MIROC6	International Centre for Earth Simulation	50
MRI-ESM2-0	Meterorological Research Institute	5
NorESM2-LM	Norwegian Climate Centre	3

Supplementary Table 3: CMIP6 models and number of realizations used in the analysis. Daily maximum temperature from the historical, historical-nat, and ssp245 experiments are used from all models.

	Dependent variable: Regional lnGDPpc
	(1)
Country lnGDPpc	1.0164***
	(0.048)
Luminosity	0.1041***
	(0.027)
Country lnGDPpc × luminosity	−0.009**
	(0.003)
Observations	24502
Clustering	Country
Adjusted R^2	0.866

$^{***}p < 0.001$; $^{**}p < 0.01$; $^{*}p < 0.05$

Supplementary Table 4: Parameters used in predicting regional GDPpc (main text Eqn. 2). Standard errors are clustered at the country level to account for spatiotemporal autocorrelation in regional growth.

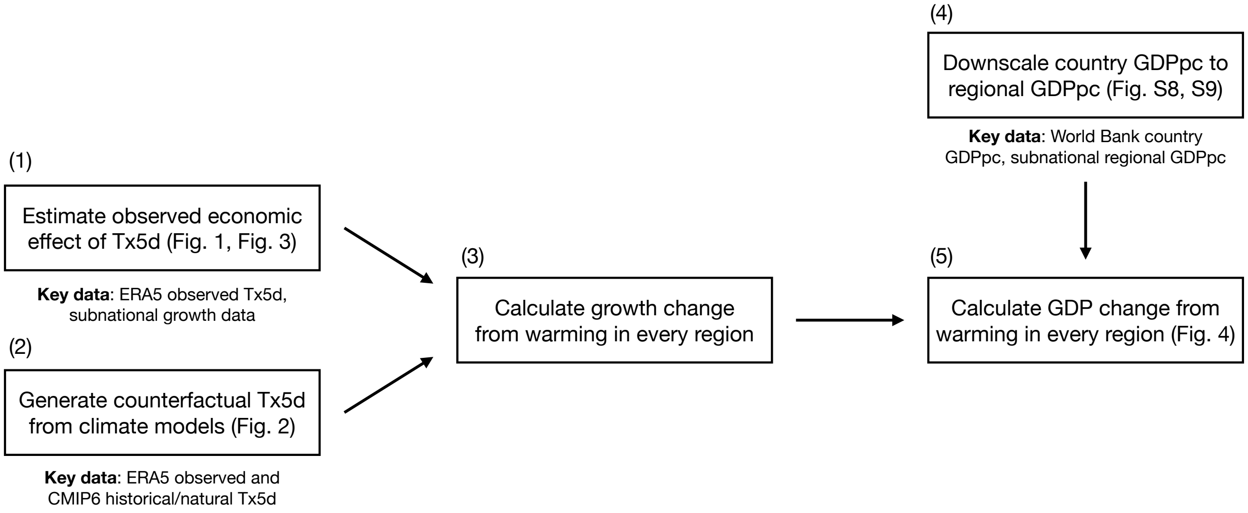

Supplementary Figure 1: Analytical framework. (1) We first estimate the effect of Tx5d intensity on economic growth using ERA5 reanalysis data and subnational growth observations (see Fig. 1 and Fig. 3). (2) In parallel, we estimate the effect of historical anthropogenic climate change on Tx5d to date using CMIP6 historical and natural simulations (see Fig. 2). (3) We then calculate regional growth changes by applying the econometric regression coefficients to the observed and counterfactual Tx5d data. Then, after (4) downscaling country income to subnational income (see Fig. S8 and Fig. S9), we (5) apply the growth change values to regional GDPpc time series to calculate observed and counterfactual GDP in each region, and thus economic damage from climate change (see Fig. 4).

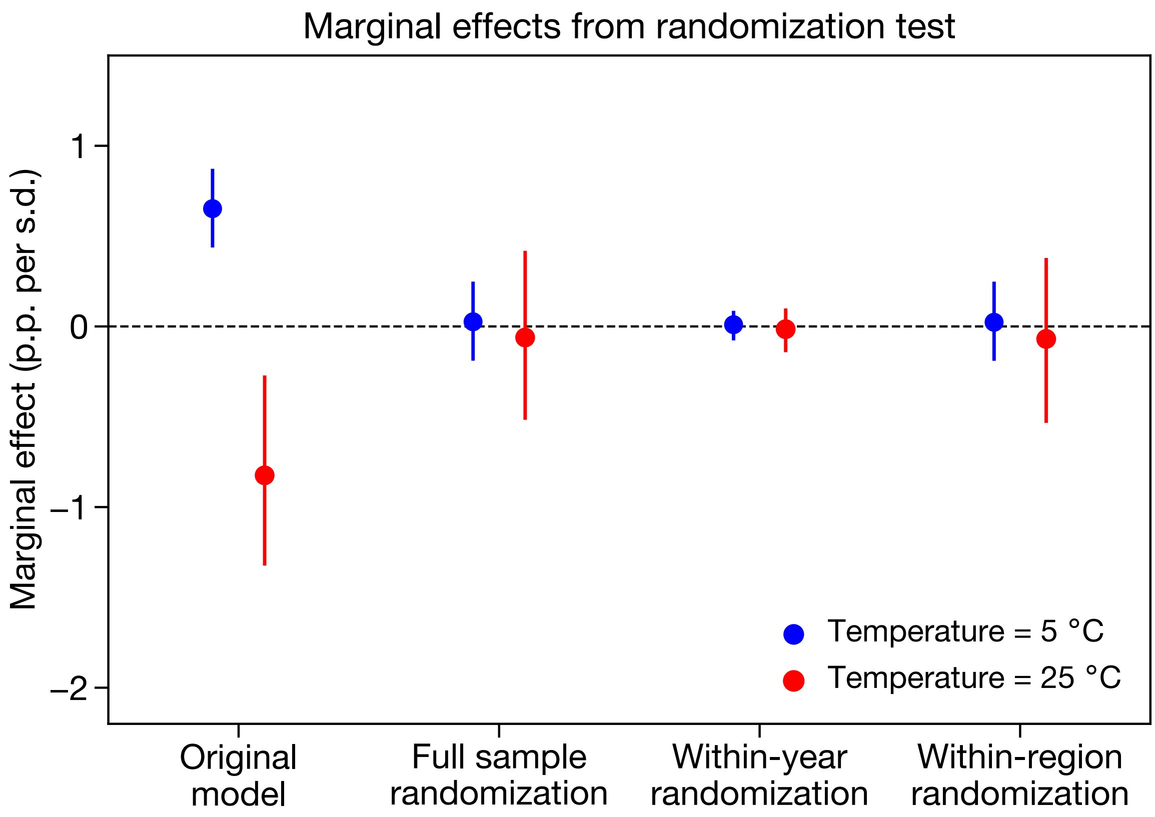

Supplementary Figure 2: Marginal effects of extremes from randomization tests. Example marginal effects of extreme temperatures on economic growth when the annual average temperature is 5 °C (blue) and 25 °C (red). The first two coefficients show the mean and 95% confidence intervals from the original model, second two coefficients show the result after Tx5d exposure is randomized across the entire sample, third two coefficients show the result after Tx5d exposure is randomized within years, and last two coefficients show the result after Tx5d exposure is randomized within regions.

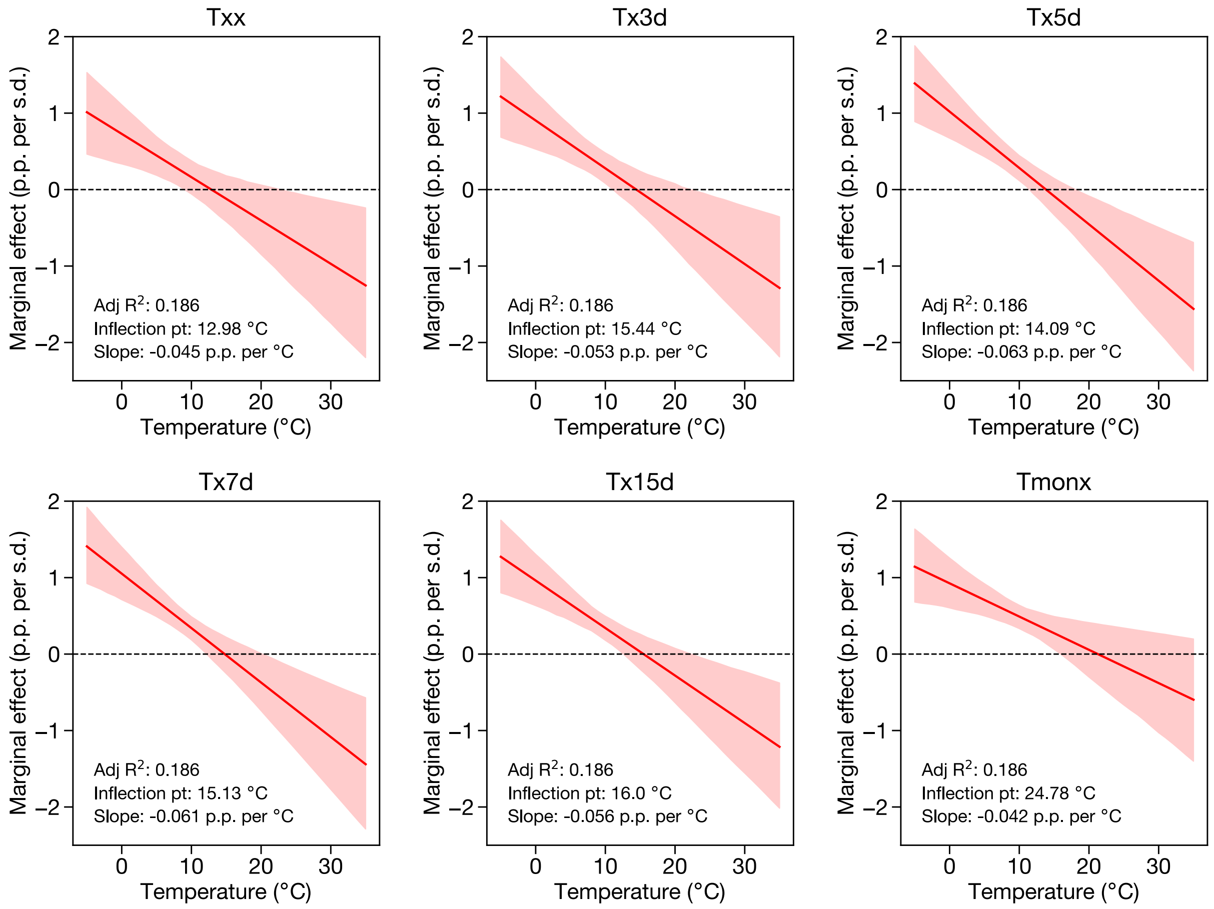

Supplementary Figure 3: Marginal effects for multiple metrics of extreme heat. Each plot shows the effect of extreme heat on regional economic growth, in p.p. per s.d., as a function of annual mean temperature. Plot axes are identical across all plots. "Txx" refers to the daily maximum temperature on the hottest day of the year, "Tx3d" refers to the average daily maximum temperature of the hottest 3-day span (and other multi-day metrics are defined accordingly), and "Tmonx" refers to the average temperature of the hottest month of the year. Solid red lines shows the mean and shading shows the 95% confidence interval across 1000 bootstrap iterations (Methods). "Adj R^2" denotes the adjusted R^2 value, "Inflection pt" denotes the average point at which the effect becomes negative across all bootstraps, and "Slope" denotes the average value of the interaction coefficient (i.e., decrease in marginal effect with increasing temperature) across all bootstraps.

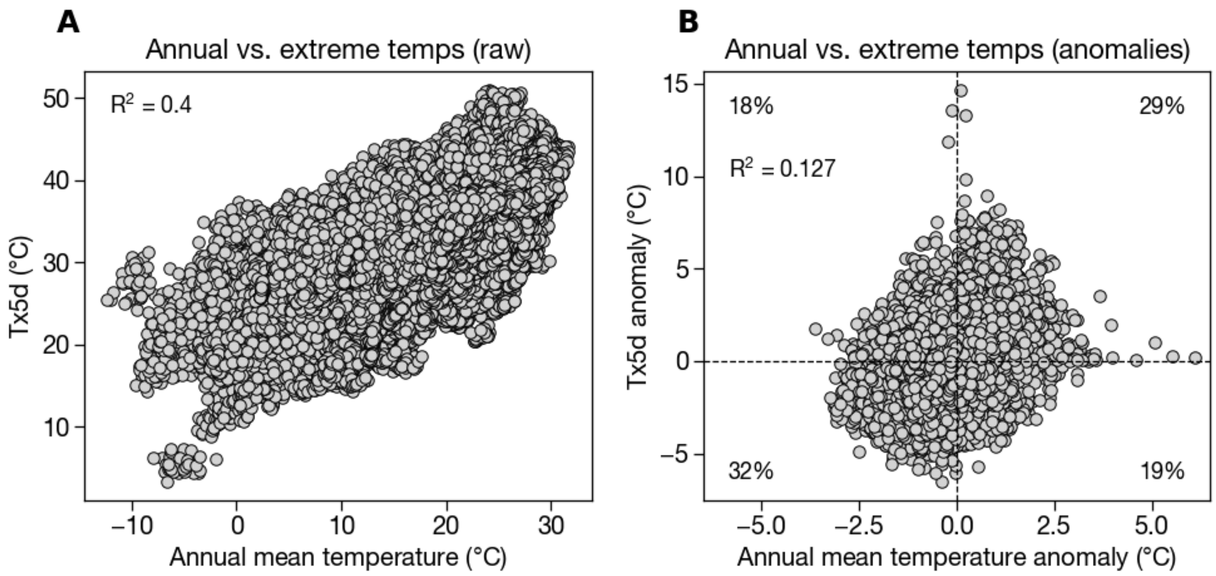

Supplementary Figure 4: Explanatory power of average temperatures for temperature extremes. A) Relationship between annual mean temperature and Tx5d, using the raw values from the full sample. B) Relationship between annual mean temperature and Tx5d when both values are calculated as deviations from regional means as would be in the estimation of a fixed effects model. R^2 in both figures refers to the R^2 value from a simple linear regression of Tx5d onto annual average temperature. Percentages in panel B denote the percent of the sample that falls into each quadrant.

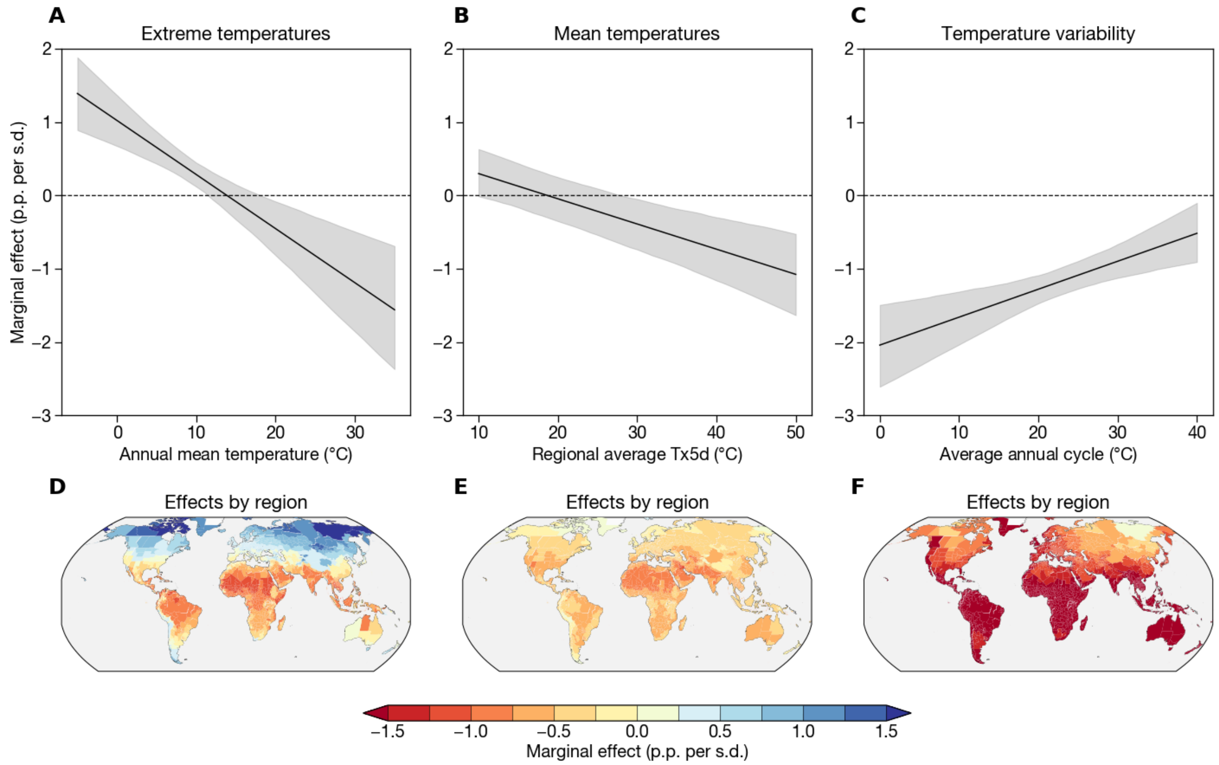

Supplementary Figure 5: Marginal effects of extreme temperatures, mean temperatures, and temperature variability. A-C) Marginal effects across annual mean temperatures for both extreme (A) and annual mean temperatures (B), and across the mean annual cycle for variability (C). Solid lines denote means and shading denotes 95% confidence intervals from bootstrap resampling (Methods). Average temperature effects are not smooth because they depend on both the annual mean temperature and Tx5d, so we calculate these effects using the average Tx5d values experienced at each annual mean temperature value (Methods). D-E) Average marginal effects in each region for extremes (D), averages (E), and variability (F). All marginal effects are standardized by the average within-region standard deviation of the relevant variable.

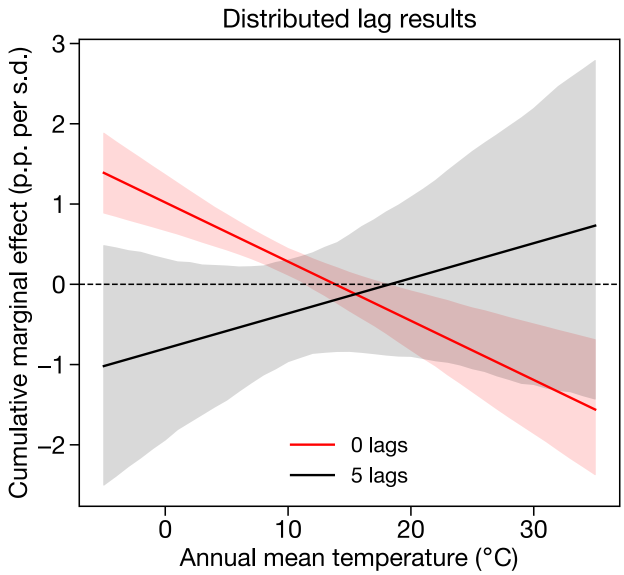

Supplementary Figure 6: Contemporaneous and cumulative effects of extreme temperatures across a range of annual mean temperatures. Red line shows the contemporaneous marginal effect and black line shows the cumulative marginal effect when 5 lagged years are added to the regression model. In both cases, the line shows the mean and the shading shows the 95% confidence intervals from bootstrap resampling, as in the main analysis.

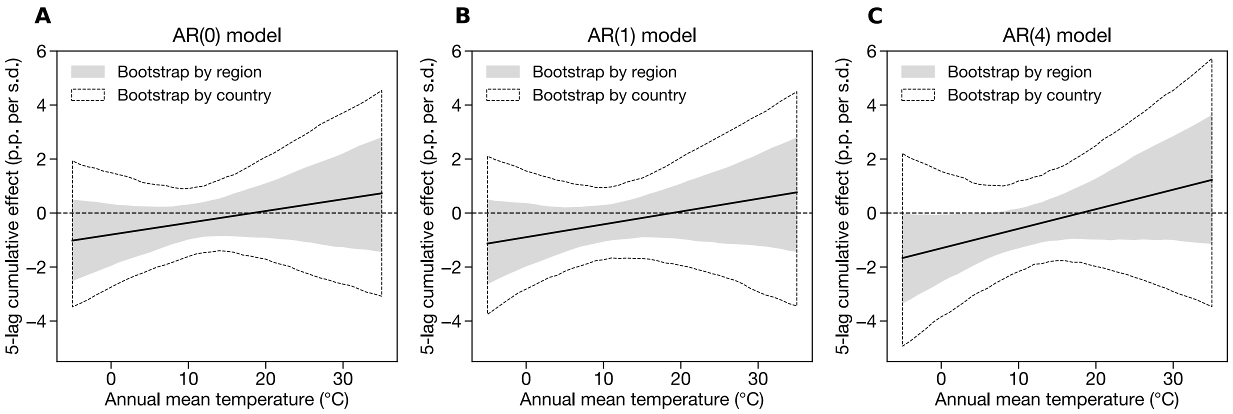

Supplementary Fig. 7: Distributed lag model results using autoregressive (AR) models. A) Cumulative 5-lag marginal effect of Tx5d increases (p.p. per s.d.) across a range of annual mean temperatures, as in the main analysis (not including an AR term). Black line shows the mean across 1000 bootstrap samples, bootstrapping by region as in the main analysis, shading shows the 95% confidence intervals (CI) when bootstrapping by region, and dashed lines show the 95% CI when bootstrapping by country. **B)** As in (A), but using an AR(1) distributed lag model, meaning that one lag of growth is added to the right-hand-side of the equation. **C)** As in (A), but using an AR(4) distributed lag model.

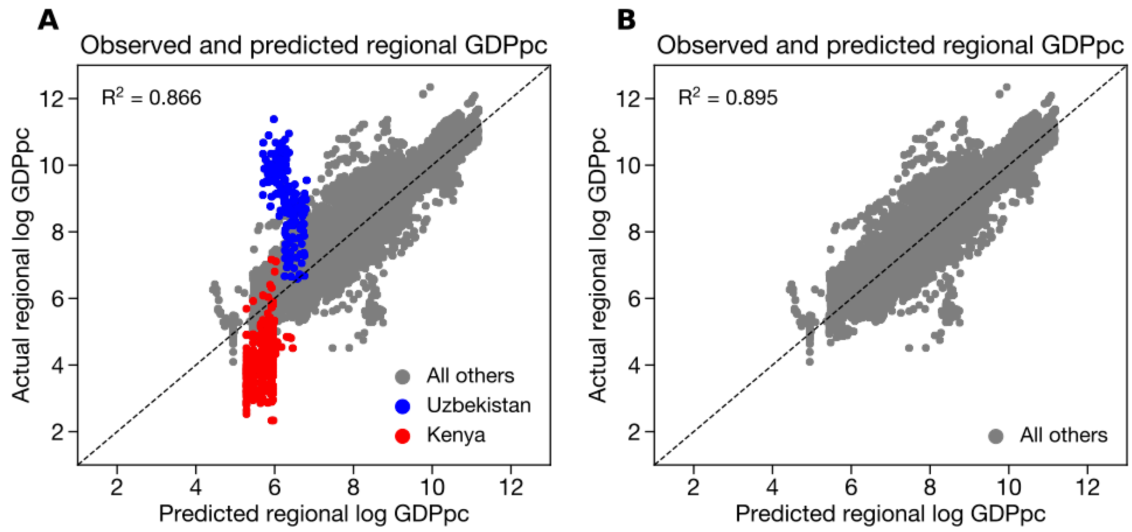

Supplementary Figure 8: Prediction of regional log GDPpc. Predicted regional log GDPpc againstactual log GDPpc when Kenya and Uzbekistan are included (A) and excluded (B). Predicted data is generated using a statistical model that includes country GDPpc and regional nighttime luminosity.

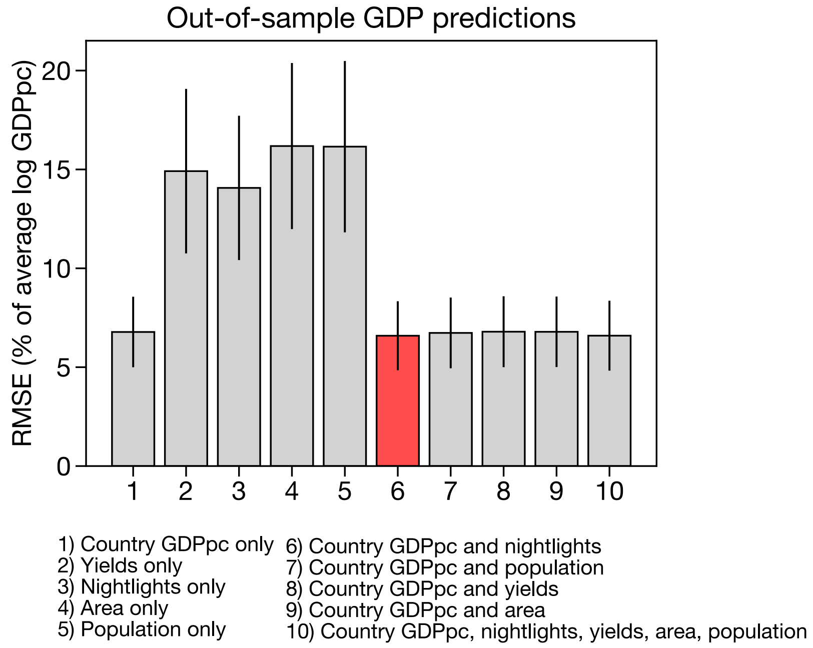

Supplementary Figure 9: Cross-validation of regional GDPpc prediction. Out-of-sample prediction error for log regional GDPpc from 10-fold cross-validation using a series of statistical models that include the parameters described in the legend. Prediction error is quantified using the root mean squared error as a percent of average regional log GDPpc. Red bar has the lowest out-of-sample prediction error. Bar heights denote means of prediction error across 10 folds and black error bars denote the mean plus or minus the standard deviation across 10 folds.

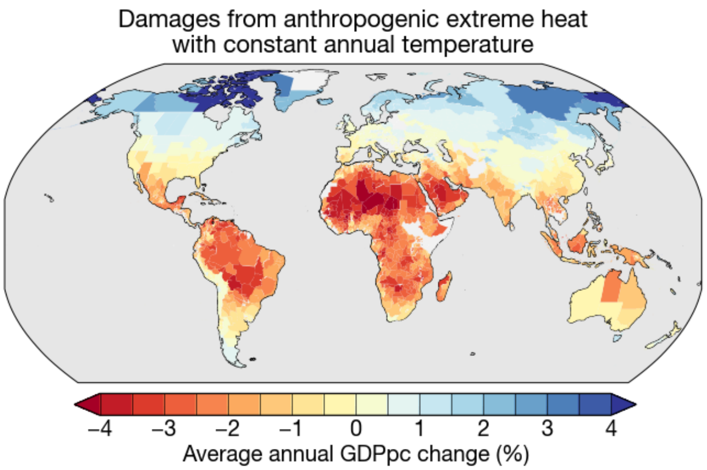

Supplementary Figure 10: Damages with constant annual mean temperature. As in Fig. 4A, but holding annual mean temperatures constant at their observed values, so the marginal effect of extremes does not change with warming.

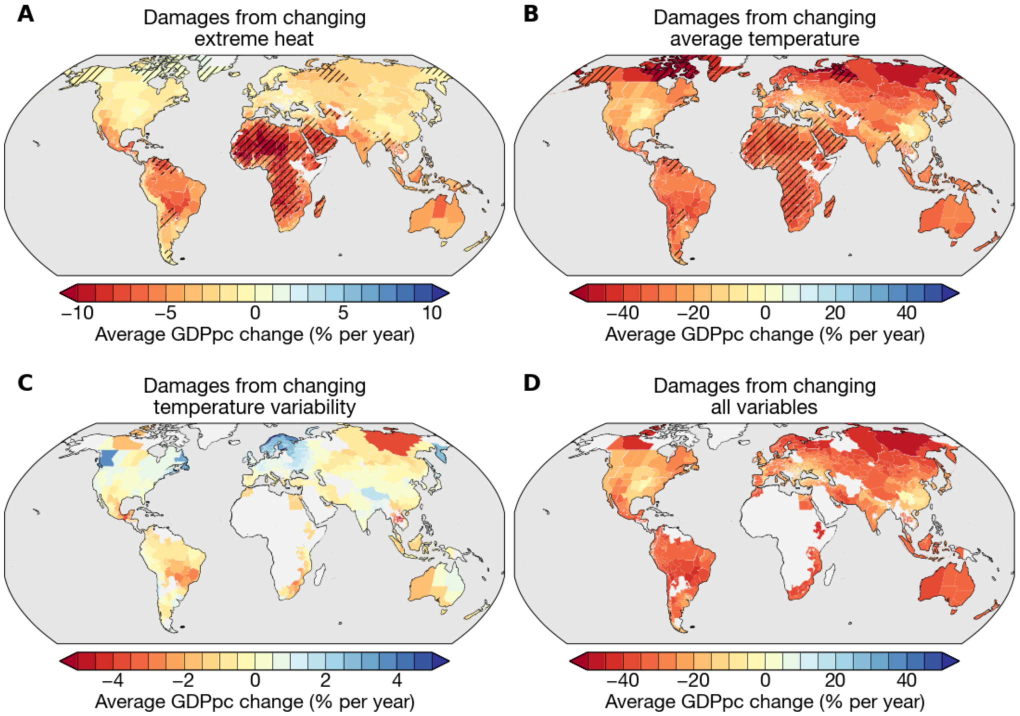

Supplementary Figure 11: Historical economic damages from changes in all temperature variables. A-D) Average change in GDP per capita due to anthropogenic changes in extreme heat intensity (A), mean temperatures (B), temperature variability (C), and the combined effects of all three variables (D). Missing data in (C) and (D) is because variability data is only available within the estimation sample. Hatched regions in (A) and (B) match the regions of missing data in (C) and (D).

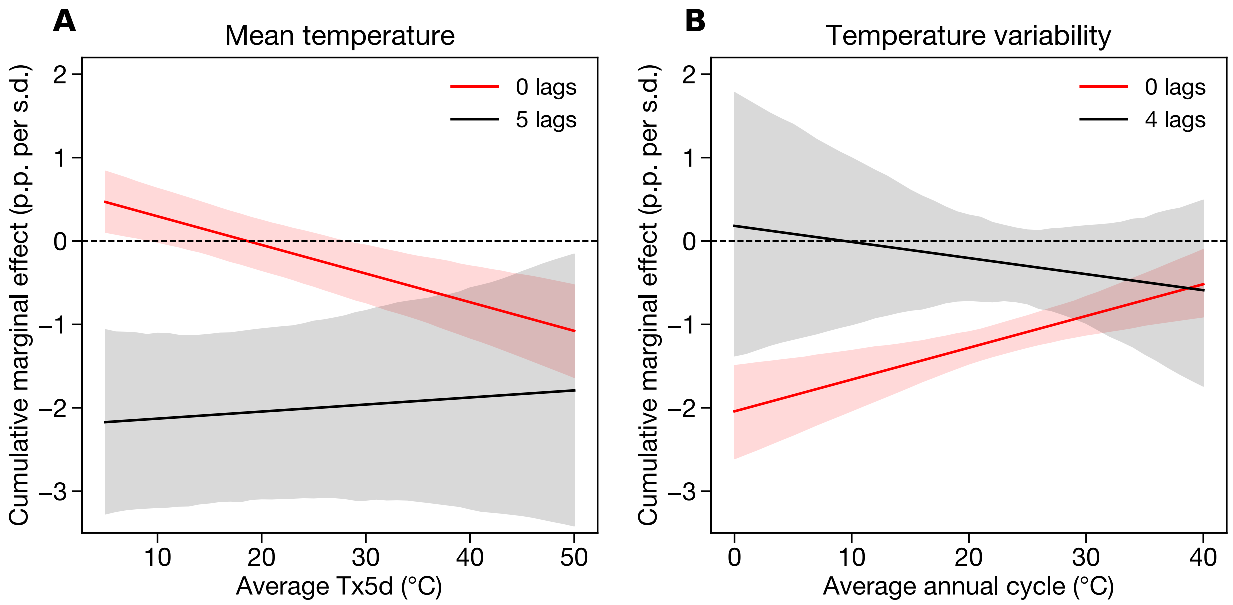

Supplementary Figure 12: Distributed lag regression estimates for average temperature and temperature variability. A) Marginal effect of a 1-s.d. increase in the annual mean temperature on regional economic growth. The contemporaneous response is shown in red and the cumulative 5-lag response is shown in black. B) Marginal effect of a 1-s.d. increase in daily-scale temperature variability on regional economic growth. The contemporaneous response is shown in red and the cumulative 4-lag response is shown in black. We choose 4 lags for panel (B) because it is the first year in which the response is indistinguishable from zero. In both panels, solid lines show average and shading shows the 95% confidence interval from bootstrap resampling (Methods).

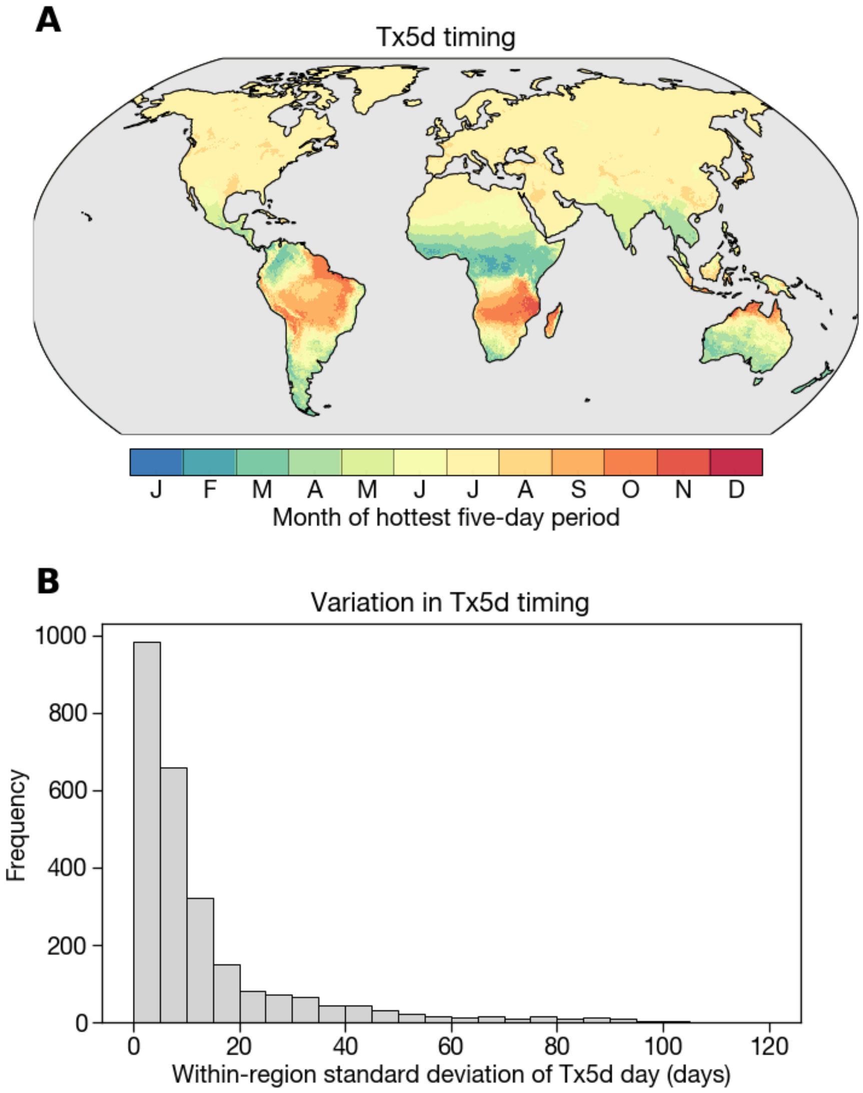

Supplementary Figure 13: Variation in Tx5d timing. A) Average month in which the center day of the hottest five-day period falls for each grid cell. **B)** Average within-region spatial standard deviation of the day of year on which the hottest five-day period is centered. A value of 10, for example, means that in an average year, the grid cells in a given region experience variation of 10 days in the timing of their hottest five-day periods.

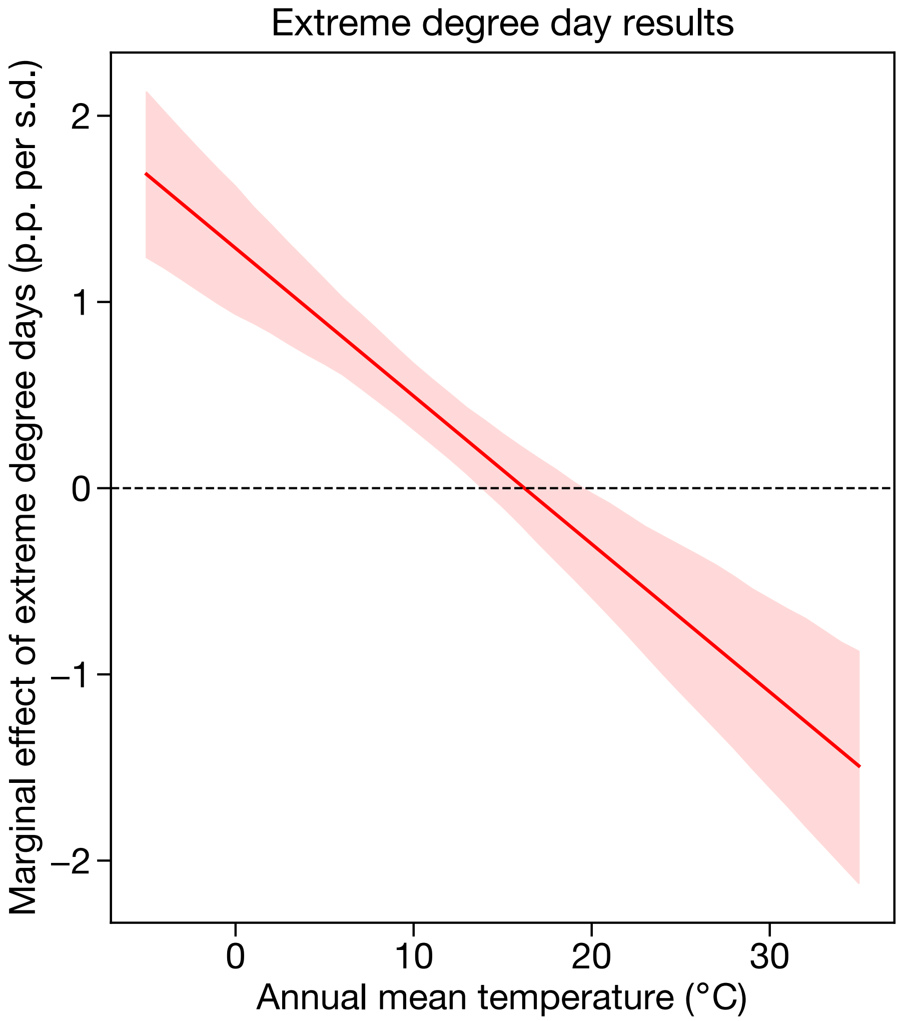

Supplementary Figure 14: Effect of extreme degree days on economic growth. Effect of increases in extreme degrees on economic growth in percentage points per standard deviation. Extreme degree days are calculated as cumulative annual temperature exposure above location- and month-specific percentile thresholds (see Supplementary Methods). Solid line shows the mean and shading shows the 95% confidence interval using bootstrap resampling, as in the main analysis.
